# Lipid, fatty acid, carnitine- and choline derivative profiles in rheumatoid arthritis outpatients with different degrees of periodontal inflammation

**DOI:** 10.1038/s41598-021-84122-y

**Published:** 2021-03-05

**Authors:** Kathrin Beyer, Stein Atle Lie, Bodil Bjørndal, Rolf K. Berge, Asbjørn Svardal, Johan G. Brun, Anne Isine Bolstad

**Affiliations:** 1 Department of Clinical Dentistry, Faculty of Medicine, University of Bergen, Årstadveien 19, 5009 Bergen, Norway; 2 Department of Clinical Science, University of Bergen, Bergen, Norway; 3 Department of Sport, Food and Natural Sciences, Western Norway University of Applied Sciences, Bergen, Norway; 4 Department of Heart Disease, Haukeland University Hospital, Bergen, Norway; 5 Department of Rheumatology, Haukeland University Hospital, Bergen, Norway

**Keywords:** Rheumatic diseases, Chronic inflammation, Periodontitis

## Abstract

Rheumatoid arthritis (RA) and periodontitis are chronic inflammatory diseases with several pathogenic pathways in common. Evidence supports an association between the diseases, but the exact underlying mechanisms behind the connection are still under investigation. Lipid, fatty acid (FA) and metabolic profile alterations have been associated with several chronic inflammatory diseases, including RA and periodontitis. Mitochondria have a central role in regulating cellular bioenergetic and whole-body metabolic homeostasis, and mitochondrial dysfunction has been proposed as a possible link between the two disorders. The aim of this cross-sectional study was to explore whole-blood FA, serum lipid composition, and carnitine- and choline derivatives in 78 RA outpatients with different degrees of periodontal inflammation. The main findings were alterations in lipid, FA, and carnitine- and choline derivative profiles. More specifically, higher total FA and total cholesterol concentrations were found in active RA. Elevated phospholipid concentrations with concomitant lower choline, elevated medium-chain acylcarnitines (MC-AC), and decreased ratios of MC-AC and long-chain (LC)-AC were associated with prednisolone medication. This may indicate an altered mitochondrial function in relation to the increased inflammatory status in RA disease. Our findings may support the need for interdisciplinary collaboration within the field of medicine and dentistry in patient stratification to improve personalized treatment. Longitudinal studies should be conducted to further assess the potential impact of mitochondrial dysfunction on RA and periodontitis.

## Introduction

Rheumatoid arthritis (RA) and periodontitis are chronic destructive inflammatory diseases that have complex multifactorial pathologic processes in common^1–4^. Current evidence supports an association between periodontitis and RA, in a to date unidentified subgroup of patients, which most likely share genetic and environmental risk factors^5^. Recent studies and meta-analysis report a higher occurrence of RA in patients with moderate to severe periodontitis compared to controls, and a higher prevalence of periodontitis and periodontal destruction in RA subjects compared to non-RA subjects^6–12^. RA patients with persistent periodontal disease have shown less responsiveness to anti-tumor necrosis factor alpha (anti-TNFα) treatment, and it is reported that periodontal treatment may reduce RA disease activity^13,14^. The relationship between RA and periodontitis has been attributed to unbalanced pro- and anti-inflammatory response and persistent inflammation along with structural damage. In both diseases, cells of the adaptive and innate immune system are found to promote the secretion of pro-inflammatory cytokines, matrix metalloproteinases (MMP) and reactive oxygen species (ROS)^15–17^. Persistent inflammation in RA and periodontitis has been associated with other comorbidities, for example cardiovascular disease (CVD)^18,19^.

Mitochondrial dysfunction has been linked to numerous diseases including RA, periodontitis, neurodegenerative diseases, diabetes, cancer, and obesity^20–24^. Mitochondria have a central role in energy utilization and energy production by regulating cellular bioenergetics and metabolism and the generation of ROS^25^. Defective or insufficient mitochondrial function at the cellular level is thought to impact whole-body metabolic homeostasis^26^. During inflammatory response, innate immune cells such as polymorphonuclear leukocytes (PMN) and macrophages have the potential to extensively increase ongoing metabolism and enhance the metabolic demand for oxygen beyond the capacity of the existing resources^27^. Consequently, hypoxia arises, causing reduced mitochondrial respiration and finally resulting in excessive production of ROS and mitochondrial DNA damage^28,29^.

The assessment of metabolic changes in diseases has been used to investigate overall metabolic activity. Metabolic profiling of patients with established RA has proven to be significantly different from healthy controls^30^. Quantitative abnormalities in blood FA and lipids have been found in RA and periodontitis^30–35^.

**Figure 1:**
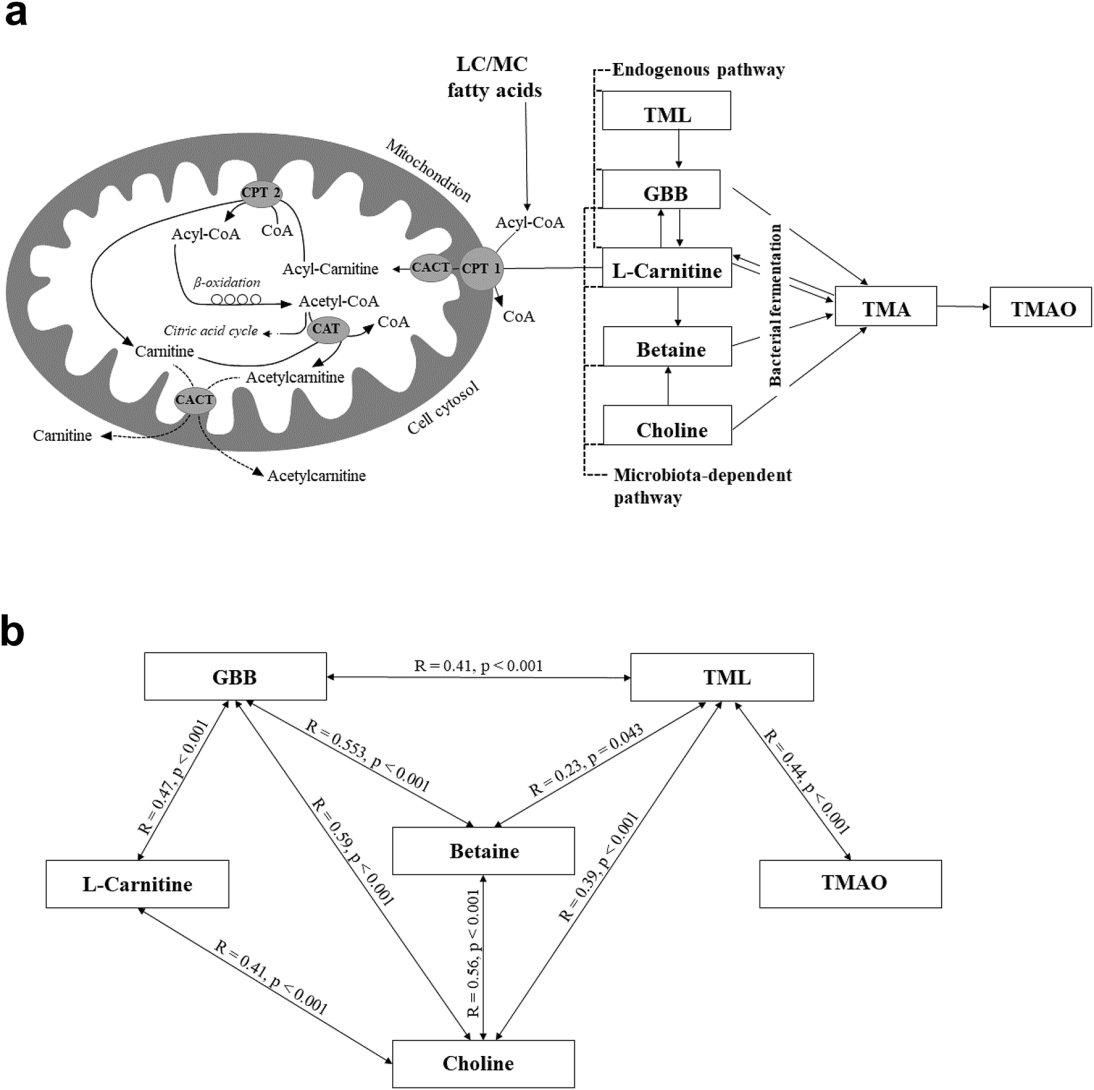
(**a**) Overview of fatty acid oxidation, carnitine biosynthesis and metabolism, and TMAO formation. (**b)** Relationship between serum carnitine- and choline derivatives in RA patients with different periodontal conditions (N = 78). CPT, carnitine palmitoyl-transferase; CACT, carnitine-acylcarnitine translocase; TML, trimethyl lysine; GBB. γ-butyrobetaine; TMA, trimethylamine; TMAO, trimethylamine N-oxide.

The naturally occurring amino acid derivative l-carnitine (levocarnitine, LC) is required to transfer medium- and long-chain FA across the inner mitochondrial membrane for subsequent β-oxidation. The plasma acetylcarnitine (AC2) level is influenced by inhibitors to FA oxidation in mice^36^. Increased acylcarnitine (AC) concentration has been linked to the progression of several chronic diseases, including insulin resistance and CVD^37,38^. LC is also found to serve as precursor for the generation of trimethylamine-N-oxide (TMAO) via a multistep metaorganismal gut microbiota-dependent pathway^39^ (Fig. 1A). TMAO is recognized to induce inflammation and to interfere with cholesterol metabolism. Elevated TMAO concentrations were found to be both proatherogenic and associated with CVD risks^40^.

The mechanisms behind a possible link between RA and periodontal disease are still not adequately elucidated. Detailed knowledge about the shared inflammatory processes will increase the understanding of the biological mechanisms behind the association. Therefore, the aim of this study was to explore whole-blood FA, serum lipid composition, carnitine- and choline derivatives including TMAO in RA patients with different degrees of periodontal inflammation to reveal any variations in RA disease activity status and periodontitis severity.

## Material and methods

### Study design

The data for this cross-sectional study were collected between May 2013 and March 2016 at the Department of Rheumatology, Haukeland University Hospital, Bergen, and Department of Clinical Dentistry, University of Bergen, Norway. Study protocol and informed consent, according to the Helsinki Declaration of 1975, version 2008^41^, were approved by the Regional Committee for Medical and Health Research Ethics (REC, Health Region West) (2012/2212), University of Bergen, Norway. All participants read and signed the written informed consent.

### Study population

In this study, RA outpatients with chronic established RA were invited to participate. RA disease was classified using the 2010 classification criteria of American College of Rheumatology/European League Against Rheumatism (ACR/EULAR)^42^. Inclusion criteria were chronic established RA, Caucasian ethnicity and ≥ 35 years of age. The criteria for exclusion were diabetes, malignancy, pregnancy, breastfeeding, and antibiotic use within 3 months prior to the study. Demographic and behavioral characteristics were collected using questionnaires. Past medical history, clinical and laboratory data on RA status and medication were obtained from medical records. RA patients were examined intra-orally and periodontal data were collected under standardized conditions by a single calibrated dentist (KB). A detailed description is provided in Beyer et al. 2018^43^.

In brief, patient-related data including RA disease debut and duration, modified health assessment questionnaire (MHAQ)^44^, RA disease activity score (DAS28-ESR)^45,46^, joint damage, and patient global health assessment scored on a visual analogue scale (VAS)^47^ were obtained. Routine laboratory analyses included erythrocyte sedimentation rate (ESR), C-reactive protein (CRP), rheumatoid factor (RF) and anti-citrullinated protein antibody (ACPA). Active RA was defined as DAS28 ≥ 2.6 and RA disease in remission as DAS28 < 2.6^48^.

A comprehensive periodontal examination including registration of pocket depth (PD), clinical attachment level (CAL), bleeding on probing (BoP) and accumulation of dental plaque (PI) at six sites per tooth was assessed. A detailed description of periodontal measurements is provided in Beyer et al.^49^. The patients were asked not to eat, drink and smoke for at least two hours prior to examination.

The assessment of periodontal status was adapted from the Centers for Disease Control (CDC)/American Academy of Periodontology (AAP) clinical case definitions^50^. Subjects were classified into three sub-groups: (1) gingivitis: PD ≤ 3 mm and BoP, (2) mild/moderate periodontitis: ≥ 2 interproximal sites with CAL 3–5 mm (not on the same tooth) or ≥ 2 interproximal sites with PD ≥ 4 mm (not on the same tooth) and BoP, or (3) severe periodontitis: ≥ 2 interproximal sites with CAL ≥ 6 mm (not on the same tooth) and ≥ 1 interproximal site with PD ≥ 5 mm and BoP.

Furthermore, subjects with measurements of CAL ≤ 5 mm, and CAL > 5 mm at least at two tooth sites were assigned into two separate groups.

Current smokers were defined as subjects who smoked or stopped smoking less than 12 months prior to study enrollment. Former smokers were subjects who quit smoking more than 12 months ago.

Peripheral venous blood samples were obtained by venipuncture at the antecubital fossa (Vacutainer Blood Collection Set, BD Vacutainer, Becton, Dickinson and Company, Franklin Lakes, NJ, USA) using 3 mL K2EDTA Vacuettes and Serum Sep Clot Activator vacutainers (Greiner Bio-One Gmbh, Kremsmuenster, Austria). Whole blood samples for FA analysis were kept on ice, frozen at  − 20 °C (within 30 min) for 24 h, then stored at  − 80 °C until analysis. Whole blood samples used for serum extraction were set to coagulate for minimum 30 min (maximum 60 min). After centrifugation at 1300× g for 10 min, the supernatant was stored at − 80 °C until analysis.

### Analysis of fatty acids by gas chromatography

Whole blood content and composition of 38 FA were analysed at the Institute of Marine Research (IMR), Bergen, Norway using ultrafast gas chromatography (UFGC) (Thermo Electron Corporation, Massachusetts, USA)^51^ as an accredited laboratory method (NS-EN ISO/IEC 17025). The FA composition was calculated using an integrator (Chromeleon 6.80, Dionex Corporation, California, USA) connected to the UFGC^52^. A standard mixture of methyl esters (Nu-Chek, Minnesota, USA) was used to ascertain the identification of FA. The responsiveness of the detector was routinely checked against the composition of a commercial mixture of FAME standards.

The absolute and relative concentrations of whole blood tissue FA were expressed as mg FA/g sample (wet weight) and as weight percentage (wt%) of total FA methyl esters (FAME), respectively. The detection limit was 0.003 mg FA/g sample, and the limit of quantification (LOQ) was 0.01 mg FA/g samples. FA below LOQ were excluded from the analysis in all patients. The concentrations of identified and unidentified FA were summed up to the total amount of measured FA. For FA profile data analysis, the relative concentration of each FA (wt%) over the sum of all FA (mg/g) was calculated. FA were described by their lipid structure using the C:Dn−x nomenclature. Common FA names have been enclosed for more convenient identification. FA were divided according to their chemical classification into saturated FA (SFA), monounsaturated FA (MUFA), polyunsaturated FA (PUFA), and sum n−3 and n−6 PUFA. Further, the following ratios and indices were calculated in wt%: n−3/n−6, AA/EPA, AA/LA, EPA/ALA, omega-3 index (EPA + DHA), anti-inflammatory fatty acid (AIFA) index [(EPA + dihomo-gamma-linolenic acid (DGLA) + DHA + DPA)/AA)*100), and de novo lipogenesis (DNL) index (C16:0/LA). Omega-3 index data were categorized into tertiles (lowest, middle, and highest tertile). Additionally, desaturase and elongase (Elo) activity indices have been calculated as percentages of individual FA product/precursor ratio: Elo activity from C16:0 to C18:0: C18:0/C16:0; Δ5-desaturase (D5D) for n−6 FA: AA/DGLA. D5D for n−3 FA was not evaluated since the LOQ for stearidonic acid (SDA) was reached in only five patients.

### Analysis of serum lipids

Serum lipid samples were measured enzymatically on a Hitachi 917 system (Roche Diagnostics GmbH, Mannheim, Germany) using the triacylglycerol (TG; GPO-PAP), cholesterol (CHOD-PAP), high density lipoprotein cholesterol (HDL-C)/low-density lipoprotein cholesterol (LDL-C) plus kits from Roche Diagnostics. The non-esterified FA (NEFA) and phospholipids (PL) were measured using the NEFA FS kit and Phospholipids FS kit from Diagnostic Systems GmbH (Holzheim, Germany), respectively. The results are expressed as mmol/L sample. The following ratios and indices have been calculated: HDL-C/LDL-C, Non-HDL-C [total cholesterol (TC) minus HDL-C], Castelli`s risk index (TC/HDL-C)^53^ and atherogenic index [log10(triglyceride, TG)/HDL-C]^54^. Cut-offs for dyslipidemia were set as follows: TG > 2.6 mmol/L; TC > 6 mmol/L; HDL-C < 1 mmol/L or LDL-C > 4 mmol/L.

### Analysis of serum carnitine- and choline derivative

Serum choline, betaine, trimethyl lysine (TML), γ-butyrobetaine (GBB) and TMAO, as well as free l-carnitine and seven AC were analyzed using liquid chromatography-tandem-mass spectrometry (LC–MS/MS)^55,56^. The following AC grouped by chain length were measured: the short chain (SC)-AC: AC2, propionylcarnitine (AC3), iso-/L-valerylcarnitine (AC5); the MC-AC: octanoylcarnitine (AC8), lauroylcarnitine (AC12); and the LC-AC, including myristoylcarnitine (AC14) and palmitoylcarnitine (AC16). The concentration of the serum metabolites is expressed in $\mu$ M. The following acylcarnitine ratios AC2/AC8, AC2/AC12, AC2/AC14, AC2/AC16, were calculated. Further, TMAO values were categorized into percentiles (lowest and highest percentile).

### Statistical analyses

Statistical analyses were performed using Stata SE 15.0 for Microsoft Windows (Stata Corp LP, Texas, USA). Graphs were designed with GraphPad Prism 7, version 7.04 for Microsoft Windows (GraphPad Software, La Jolla, California, USA). All continuous and categorical data were expressed as mean ± standard deviation and as number (percentage) of patients respectively, if not explicit stated differently.

Continuous numeric outcomes were tested for logarithmic (log) normality. Since no statistically relevant differences were found between log- and non-log transformed data, statistical analyses were conducted on non-log transformed data using Person chi-square, Student t-test and one-way analysis of variance (ANOVA). In the ANOVA, Scheffe`s post hoc test was conducted to determine possible group differences. Pearson’s correlation coefficient was used to measure the statistical relationship between selected continuous variables. A *P*-value less than 0.05 was considered statistically significant.

## Results

The study population consisted of RA patients (N = 78, 73% females) aged 57 ± 12 years with different degrees of periodontal inflammation. The sociodemographic and clinical characteristics of the study population are described in detail in Table 1, and Beyer et al.^43,49^. Further patient characteristics have been described and categorized by prednisolone medication in Supplementary Table S3, by periodontal status in Supplementary Table S4, and CAL in Supplementary Table S5.

**Table 1:** Patient characteristics grouped by rheumatoid arthritis (RA) disease activity (DAS 28).

	Total N = 78	RA remission N = 33	Active RA N = 45	*P* value
Females (N/%)	57/73	24/73	33/73	0.95
Age (years)	57 ± 12	57 ± 12	58 ± 12	0.70
BMI (kg/m^2^)	26.1 ± 4.3	25.9 ± 4.4	26.3 ± 4.4	0.67
**Smoking status (N/%)**				
Never smokers	29/37	12/36	17/38	0.74
Former smokers	35/45	17/52	18/40	
Current smokers	14/19	4/12	8/22	
**Oral data**				
Number of teeth	25 ± 5	25 ± 4	24 ± 6	0.22
PD (mm)	2.8 ± 0.4	2.7 ± 0.3	2.8 ± 0.4	0.65
CAL (mm)	3.1 ± 0.8	3.0 ± 0.7	3.1 ± 0.8	0.57
BoP (% of sites)	31 ± 16	33 ± 14	30 ± 17	0.49
PI (% of sites)	33 ± 16	36 ± 19	31 ± 14	0.20
**Periodontitis (N/%)**				
No	14/18	5/15	9/20	0.68
Mild/moderate	50/64	23/70	27/60	
Severe	14/18	5/15	9/20	
**RA data**				
RA debut (age)	43 ± 14	41 ± 13	45 ± 15	0.30
RA duration (years)	15 ± 11	16 ± 12	14 ± 11	0.29
DAS28 (score)	3.0 ± 1.1	2.1 ± 0.3	3.7 ± 1.0	< 0.001
VAS (score)	28 ± 21	17 ± 14	36 ± 21	< 0.001
MHAQ (score)	0.37 ± 0.38	0.17 ± 0.25	0.52 ± 0.39	< 0.001
RF, ACPA (N/%)	60/77	28/85	32/71	0.16
**Laboratory data**				
ESR (mm/hr)	19.8 ± 15.5	12.7 ± 8.3	25.0 ± 17.5	< 0.001
CRP (mg/L)	8.8 ± 13.5	5.0 ± 10.0	11.6 ± 15.1	0.031
RF (IU/ml)*	184 ± 385	175 ± 364	190 ± 405	0.87
TG (mmol/L)	1.20 ± 0.82	1.12 ± 0.65	1.26 ± 0.92	0.43
TC (mmol/L)	5.52 ± 1.22	5.33 ± 1.02	5.64 ± 1.34	0.26
HDL-C (mmol/L)	1.64 ± 0.50	1.57 ± 0.44	1.69 ± 0.53	0.30
LDL-C (mmol/L)	3.29 ± 0.93	3.24 ± 0.76	3.33 ± 1.05	0.69

Categorical/continuous variables of demographic, behavioral and clinical characteristics.

RA disease activity, measured as disease activity score 28 (DAS28); Remission, RA disease in remission defined as DAS28 ≤ 2.6; Active RA disease defined as DAS28 > 2.6; N, number; Age, patient’s age in years at the time of clinical examination; *BMI* body mass index, *PD* probing depth, *CAL* clinical attachment level, *BoP* bleeding on probing, *PI* plaque index, *DAS28* disease activity score 28, *VAS* patient global health assessment score on a visual analogue scale, *MHAQ* modified health assessment questionnaire, RF, ACPA, seropositivity, positive tests for rheumatoid factor (RF) (*N* = 74) and/or anti-citrullinated protein antibody (ACPA) (*N* = 77); *ESR* erythrocyte sedimentation rate, *CRP* C-reactive protein, *TG* triglyceride, *TC* total cholesterol, *HDL-C* high-density lipoprotein cholesterol, *LDL-C* low-density lipoprotein cholesterol. Analysed with Pearson chi-square and Student t-test: *P*-level < 0.05. *n = 74.

Active RA was diagnosed in 58% of the patients. Erosive destruction of cartilage and bone and seropositivity against RF and/or ACPAs were not related to RA disease activity (DAS28). Current treatment with conventional disease-modifying antirheumatic drugs (c-DMARDs) was registered in 42%, biological DMARDs (b-DMARDs) in 14%, and c- and b-DMARDs in 39% of the patients, and 27% of the patients were treated with prednisolone. DAS28, VAS, MHAQ and the inflammation markers ESR and CRP varied significantly between active RA and RA disease in remission, whereas age, BMI, smoking status, periodontal conditions, or treatment mode with c- and/or b-DMARDs did not differ. Periodontal examination revealed severe periodontitis in 18% of the individuals, 64% had mild or moderate periodontitis and 18% were diagnosed with gingivitis. Eighteen percent of the patients were current smokers, whereas 47% were former smokers.

### Evaluation of whole blood fatty acid and serum lipid profiles

**Figure 2:**
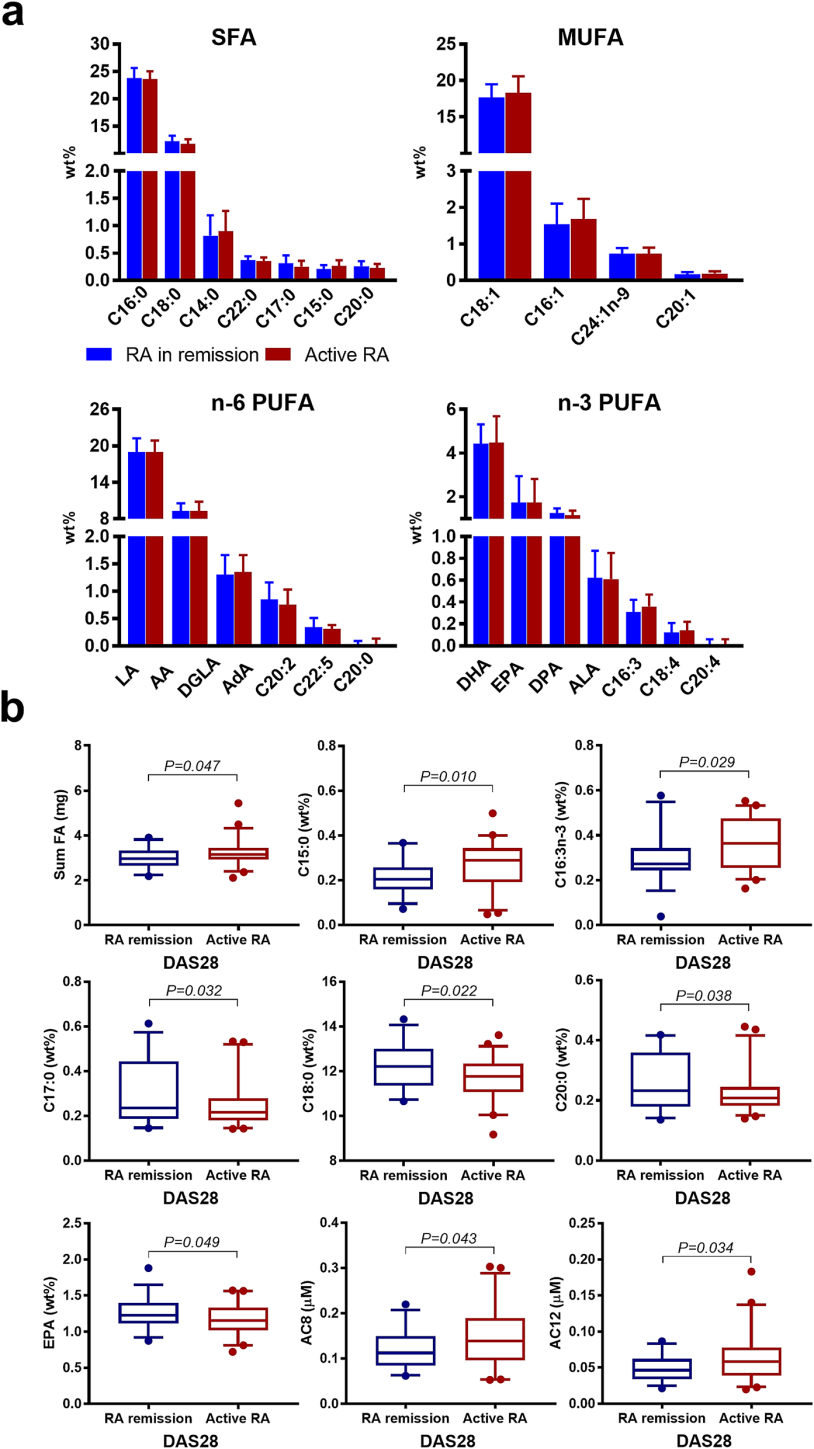
Association between whole blood FA, serum lipids, serum carnitine- and choline derivatives and RA disease activity. (**a**) Columns show relative FA concentrations (mean ± SD) grouped by RA disease activity, DAS28 score ≤ 2.6 = RA in remission (N = 33, coloured in blue) or DAS28 ≥ 2.6 = active RA (N = 45, coloured in red) and by chemical classification (SFA, MUFA, PUFA n−6, PUFA n−3) sorted from high to low abundance. (**b**) Boxplots show FA, lipids, and carnitine- and choline derivatives significant associated with RA disease activity status (RA in remission (N = 33, coloured in blue, active RA (N = 45, coloured in red). The boxplots show medians, the error bars indicate 5–95% confidence interval. The connectors show statistically significant differences, *P* < 0.05. *P *values are based on Student t-test.

Of the measured FA, 24 reached LOQ and were included into the analysis. The distribution and absolute FA concentration described as median and range are presented in Supplementary Table S1. The FA composition has been visualized in Fig. 2A grouped by RA disease status. The predominant FA species were C16:0 and C18:0 among SFA, C18:1 and C16:1 among MUFA, LA and AA among n−6 PUFA, and DHA, EPA and DPA among n−3 PUFA. Of lipids, TC, TG, HDL-C, LDL-C, NEFA and PL including the corresponding ratios and indices were analyzed.

### Assessment of fatty acid and lipid composition in relation to RA disease activity, prednisolone medication and periodontitis severity

**Figure 3:**
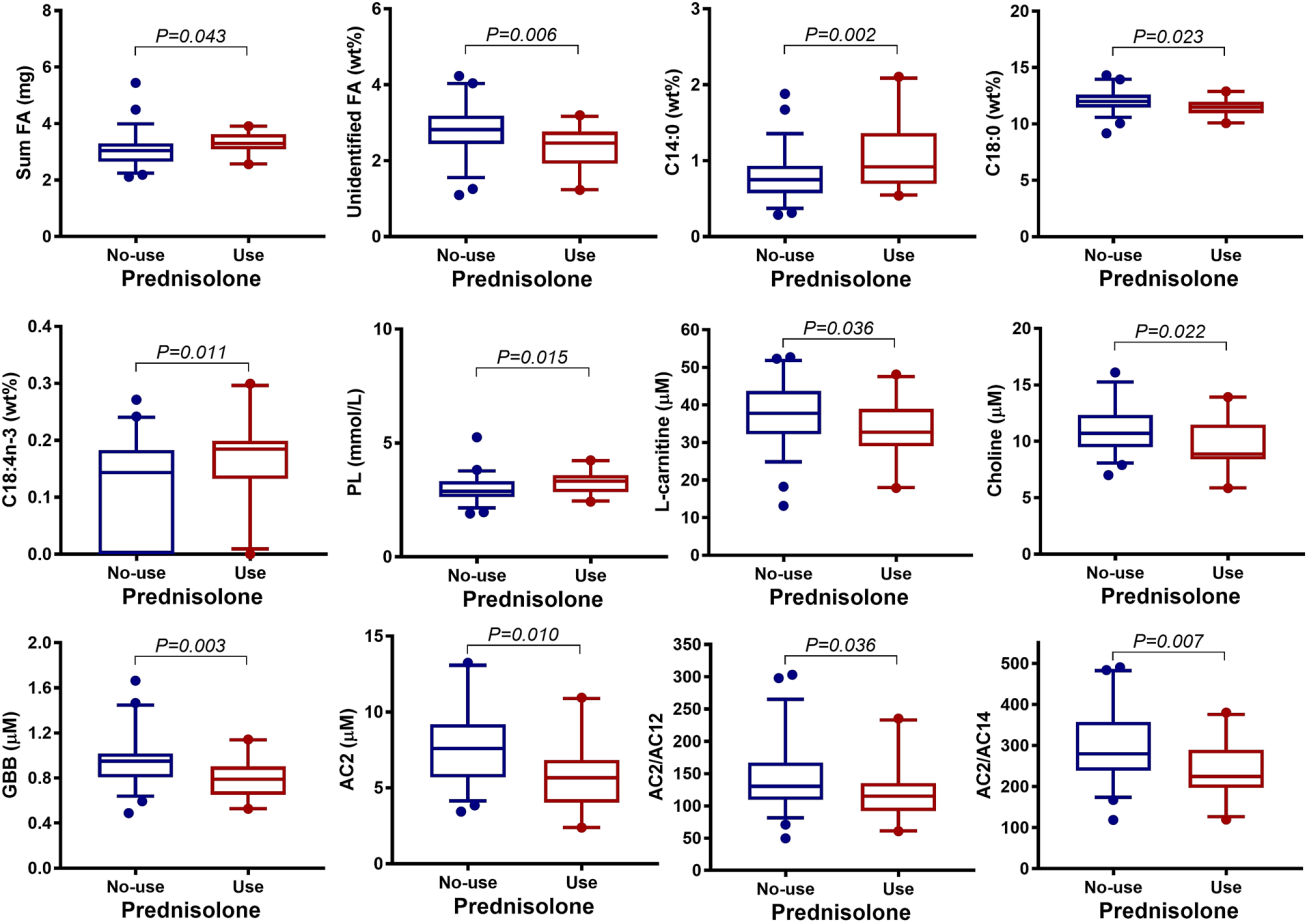
Association between whole blood FA, serum lipids, serum carnitine- and choline derivatives and prednisolone medication in RA patients (N = 78). Boxplots show FA, lipids, and carnitine- and choline derivatives significant associated with intake of prednisolone (no prednisolone, N = 57, coloured in blue; prednisolone medication, N = 21, coloured in red). *P* values are based on Student t-test. The boxplots show medians, the error bars indicate 5–95% confidence interval. The connectors show statistically significant differences, *P* < 0.05. *P *values are based on Student t-test.

**Figure 4:**
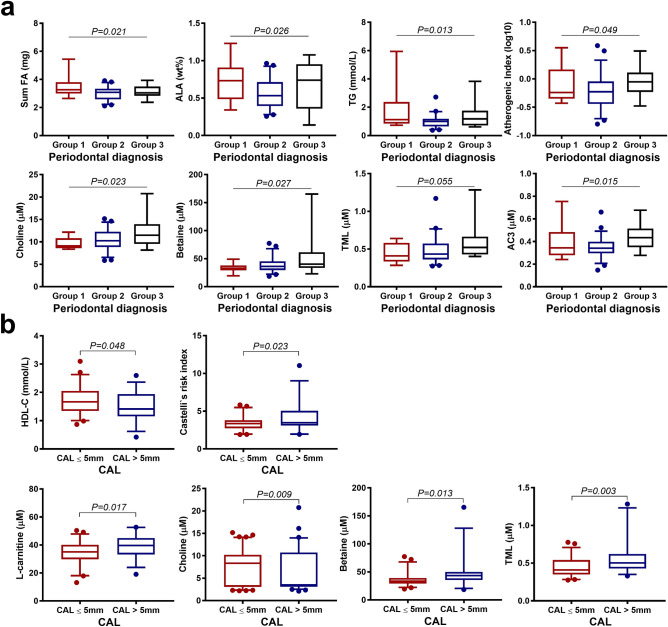
Association between whole blood FA, serum lipids, serum carnitine- and choline derivatives and periodontal conditions in RA patients (N = 78). (**a**) Boxplots show FA, lipids, and carnitine- and choline derivatives significant associated with the diagnosis of periodontal disease, grouped by severity: group 1 = gingivitis (N = 14, coloured in red), group 2 = mild/moderate periodontitis (N = 50, coloured in blue), group 3 = severe periodontitis (N = 14, coloured in black). *P*-values are based on ANOVA. The boxplots show medians, the error bars indicate 5–95% confidence interval. The connectors show statistically significant differences within these groups, *P* < 0.05). (**b**) Boxplots show lipids and carnitine- and choline derivatives significant associated with measurements of clinical attachment level, grouped by CAL ≤ 5 mm (N = 50, coloured in red) and CAL > 5 mm (N = 28, coloured in blue). The boxplots show medians, the error bars indicate 5–95% confidence interval. The connectors show statistically significant differences, *P* < 0.05. *P*-values are based on Student t-test.

The total FA concentration was higher in patients with active RA compared with patients in RA remission (*P* = 0.047) (Fig. 2b, Supplementary Table S2). Likewise, RA patients with prednisolone treatment had higher total FA concentrations compared to those without prednisolone (*P* = 0.043) (Fig. 3, Supplementary Table S3). In regard to periodontal status, the total FA concentration was highest in gingivitis and lowest in mild/moderate periodontitis (Fig. 4a, Supplementary Table S4). Allocating RA patients by CAL ≤ 5 mm and CAL > 5 mm, the FA profiles did not show any significant differences (Supplementary Table S5). None of the main FA classes categorized by their chemical classification as SFA, MUFA, PUFA differed for any of the aforementioned grouping variables. Although several individual FA varied with for RA disease activity status (Fig. 2b, Supplementary Table S2), prednisolone treatment (Fig. 3, Supplementary Table S3) or periodontal status (Fig. 4a, Supplementary Table S4), solely C15:0 was positively associated with CRP (*P* < 0.01, *R* = 0.30).

Assessment of the lipid concentrations, TG, TC, HDL-C, LDL-C and PL revealed no relation to RA disease activity status (Supplementary Table S2). RA patients taking prednisolone had a higher PL concentration compared to no prednisolone (*P* = 0.015) (Fig. 3, Supplementary Table S3). TC and HDL-C showed a slightly, but not significant, higher concentration related to prednisolone use (Supplementary Table S3).

Regarding periodontal inflammation, TG was lowest in patients with mild/moderate periodontitis and highest in patients with gingivitis. Additionally, the most unfavorable atherogenic index was found in RA patients with mild/moderate periodontitis (Fig. 4a, Supplementary Table S4). Increased CAL > 5 mm was related to a lower concentration of HDL-C (*P* = 0.048) and a more unfavorable Castelli`s risk index compared to CAL ≤ 5 mm (Fig. 4b, Supplementary Table S5). Interestingly, evaluating dyslipidemia, a more than twice as high percentage of active RA patients (42.2%) had a TC concentration > 6 mmol/L compared to RA patients in remission (18.2%, *P* = 0.025). However, despite elevated TC concentrations in approximately one third of the RA patients, elevated LDL-C and decreased HDL-C concentrations were found in only 19.2% and 6.4% of the patients, respectively.

### Fatty acid and lipid profiles in relation to smoking status

**Figure 5:**
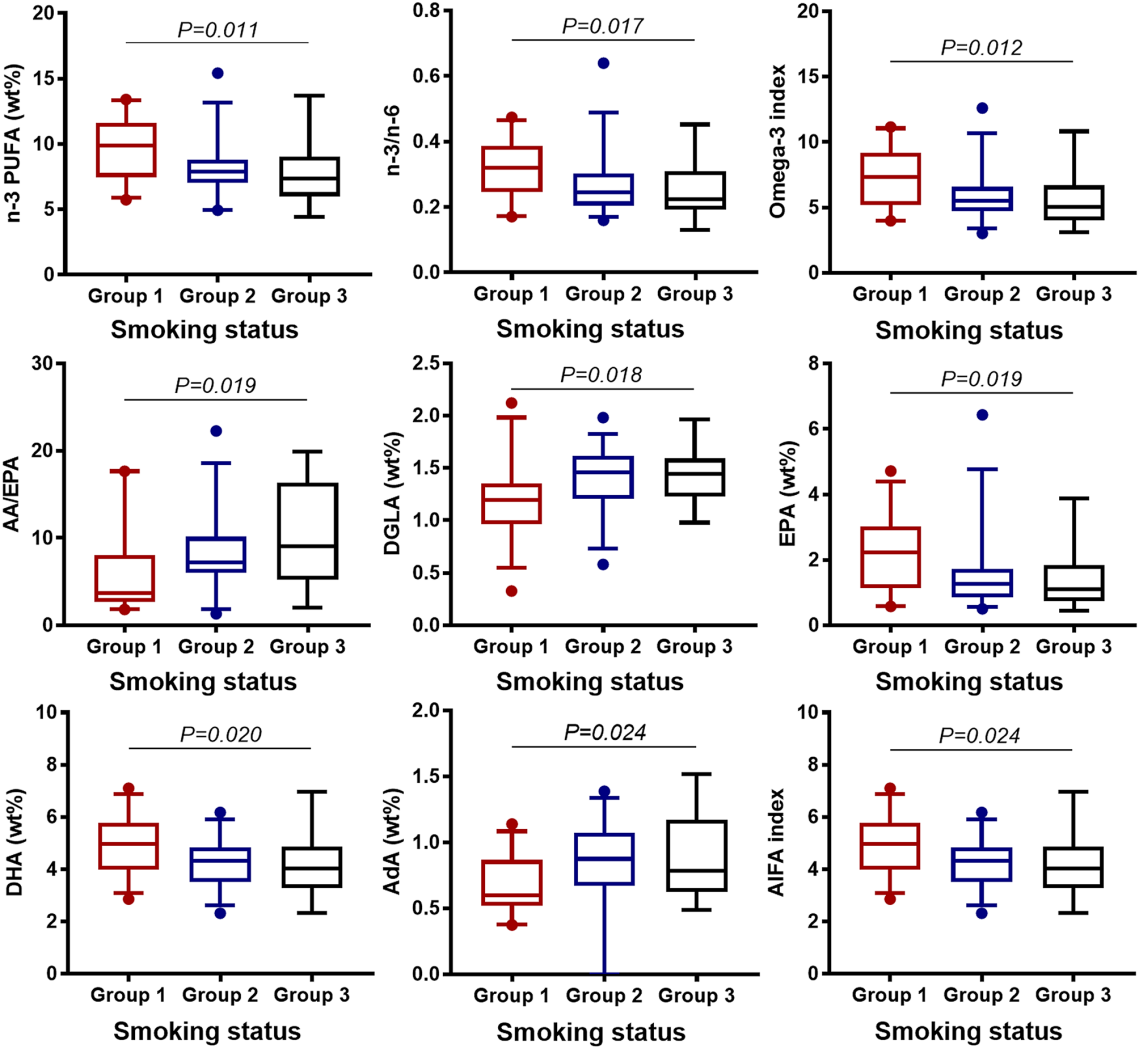
Association between whole blood FA and smoking status in RA patients (N = 78). Boxplots show FA, FA indices and -ratios significant associated with smoking status: group 1 = never smokers (N = 29, coloured in red), group 2 = former smokers (N = 35, coloured in blue), group 3 = current smokers (N = 14, coloured in black). The boxplots show medians, the error bars indicate 5–95% confidence interval. The connectors show statistically significant differences within these groups, *P* < 0.05. *P*-values are based on ANOVA.

The concentrations of n-3 PUFA, n-3/n-6, EPA, DHA, omega-3 index, AIFA increased from current, to former, to never smokers, whereas the concentration of AdA decreased. AA/EPA concentration was lowest in never smokers, elevated in former smokers and was most unfavorable in current smokers (Fig. 5, Supplementary Table S6). Lipid parameters did not differ within smoking status (Supplementary Table S6).

### Assessment of serum carnitine- and choline derivatives including trimethylamine N-oxide

Performing Pearson’s correlation, the following serum metabolites were associated: l-carnitine correlated with GBB and choline; betaine correlated with GBB, choline and TML; GBB correlated with choline and TML; choline correlated with TML; and TML correlated with TMAO (Fig. 1b). Choline as precursor of phosphatidylcholine, a major constituent of cell membranes, was negative correlated with PL (*R* =  − 0.26, *P* < 0.020). Additionally, as part of the structural components of lipoproteins, choline correlated negatively with HDL-C (*R* =  − 0.38, *P* < 0.001). Choline and betaine also correlated with CRP (*R* = 0.26, *P* = 0.020 and *R* = 0.29, *P* = 0.011, respectively), whereas none of the other serum metabolites reached the level of significance.

### Serum acylcarnitine profiles in relation to RA disease activity status and prednisolone treatment and periodontitis severity

Serum concentrations of the acylcarnitines AC8 and AC12 were higher in active RA compared to RA in remission (Fig. 2b, Supplementary Table S2). RA patients with prednisolone medication had lower AC2 concentrations compared to those without prednisolone (Fig. 3, Supplementary Table S3). Of the acylcarnitine ratios, the AC2/AC12 and AC2/AC14 decreased in patients with prednisolone medication compared to no prednisolone (Fig. 3, Supplementary Table S3). AC12 was positively related to CRP (*P* = 0.027, *R* = 0.25). Further, AC3 was highest in severe periodontitis and lowest in the mild/moderate periodontitis group (Fig. 4a, Supplementary Table S4).

### Serum carnitine- and choline derivatives in relation to RA disease activity, prednisolone medication and periodontitis severity

l-carnitine, choline, betaine, GBB, and TML did not differ for RA disease activity status (Supplementary Table S2). However, RA patients with prednisolone medication compared with no prednisolone had lower concentrations of l-carnitine, choline and GBB (Fig. 3, Supplementary Table S3). Aggravating periodontal inflammation was related to increased betaine and choline concentrations (Supplementary Table S4). RA patients with CAL > 5 mm had higher concentration of l-carnitine, choline, betaine and TML compared to patients with CAL ≤ 5 mm (Fig. 4b, Supplementary Table S5).

### Assessment of trimethylamine N-oxide

**Figure 6:**
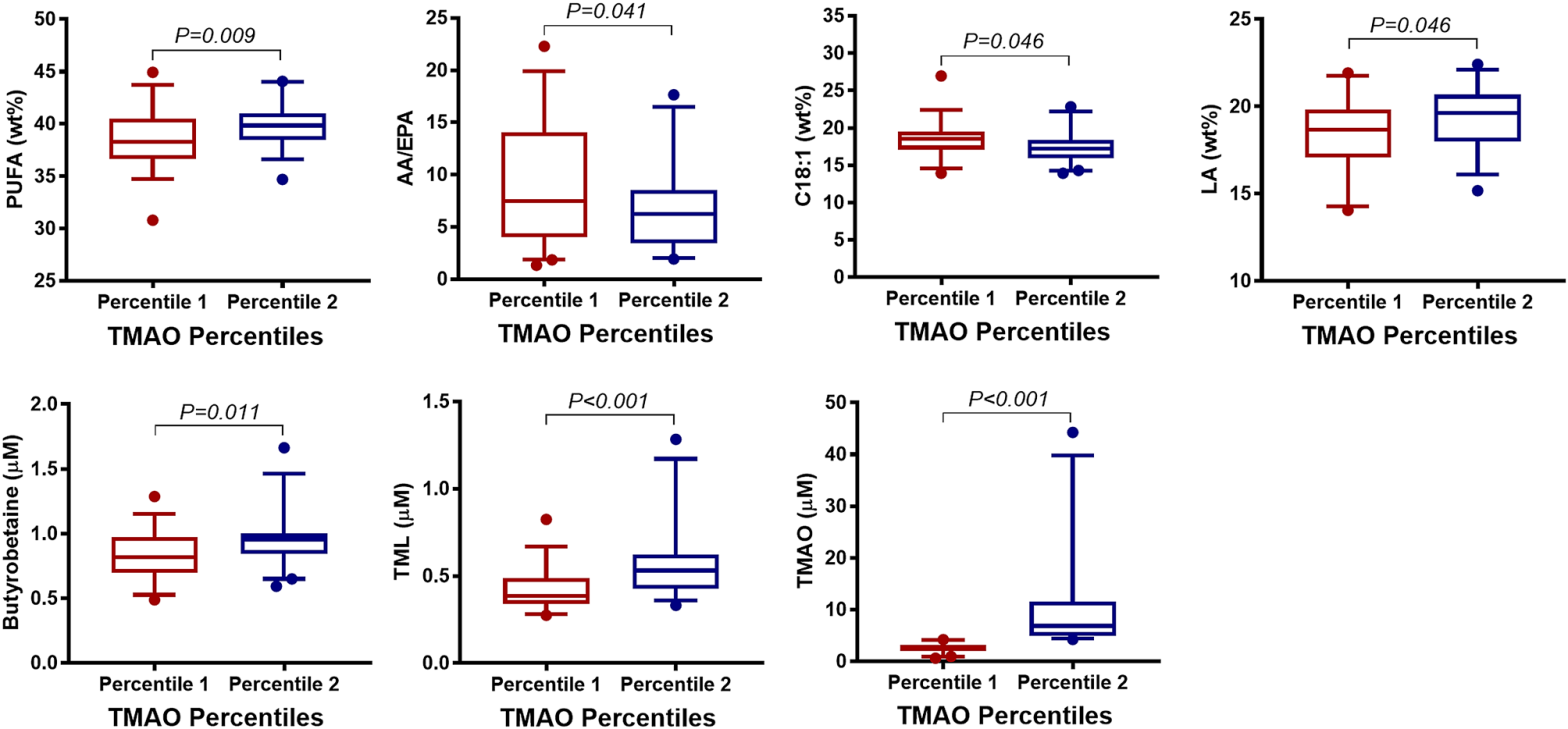
Association between whole blood FA, serum carnitine- and choline derivatives, and TMAO percentiles in RA patients (N = 78). Boxplots show FA and carnitine- and choline derivatives significant associated with TMAO percentiles, categorized into lowest TMAO percentile: percentile 1 (0.67–4.21 µM, N = 39, coloured in red) and highest TMAO percentile: percentile 2 (4.29–44.28 µM, N = 39, coloured in blue). The boxplots show medians, the error bars indicate 5–95% confidence interval. The connectors show statistically significant differences, *P* < 0.05. *P*-values are based on Student t-test.

TMAO concentration did not show significant differences for RA disease activity (Supplementary Table S2), prednisolone medication (Supplementary Table S3), periodontal diagnosis (Supplementary Table S4), CAL (Supplementary Table S5) or smoking (Supplementary Table S6). Categorizing TMAO into percentiles, in the highest percentile, the sum of PUFA, LA, the serum metabolites GBB and TML were increased, whereas AA/EPA and C18:1 were decreased, compared to the lowest percentile (Fig. 6, Supplementary Table S7). Categorizing TMAO levels by omega-3 tertiles, the highest TMAO concentration was about twofold higher in the 3rd (4.57 ± 4.06 µM) compared to the 1st tertile (10.18 ± 12.16 µM, P = 0.015) (Supplementary Table S8).

Smoking was not related to any changes in acylcarnitine, carnitine- and choline derivatives or TMAO (Supplementary Table S6).

## Discussion

This cross-sectional study shows a statistically significant relation between abnormal elevated TC in active RA compared to TC levels in RA remission. This conflicts with some former studies reporting reduced TC concentration in relation to active RA disease^57–59^. In liver mitochondria, cholesterol accumulation has been found to disrupt mitochondrial functional performance which can contribute to oxidative stress and organ injury^60^. Similarly, cultured macrophages of advanced atherosclerotic lesions are found to progressively accumulate TC, which stimulates PL biosynthesis as a response to protect the macrophages from TC-cytotoxicity. A prolongated cholesterol load has been related to mitochondrial dysfunction and cellular apoptosis^61^. Further, more recent evidence indicates that mitochondrial cholesterol-load influence mitochondrial function in several neurological diseases such as Alzheimer’s disease^62^.

Serum PL concentration was significantly higher in RA patients taking prednisolone medication compared to no prednisolone. Our finding is comparable to results of a more recent study of PL classes that observed higher total PL in synovial fluid of RA patients compared to controls^63^. Similarly, the total FA concentration was significantly higher in active RA compared to RA in remission and in patients taking prednisolone compared to no prednisolone medication. Elevated FA concentration in blood is known to increase inflammatory processes^64^ and to be increased by inflammation^65^. However, except for some specific FA, neither the main FA classes nor AIFA or DNL index were found to differ. Additionally, the analysis of serum AC revealed a higher concentration of the MC-AC in patients with active RA, and a decrease in some AC2 to MC-AC and LC-AC ratios in those of the RA patients taking prednisolone medication. Furthermore, AC12 was also found positively correlated with CRP. AC are generally considered the transport form of FA (C2-C26)^66^. Accumulation of toxic lipid product intermediates, including MC- and LC-AC have been suggested as an indicator for inefficient LC-FAO. FAO inhibition is known as a characteristic in mitochondrial dysfunction^67^.

Interestingly, the metabolite choline was negatively correlated to PL in RA patients taking prednisolone medication compared to no prednisolone. Equally to in our study, others found choline to correlate with CRP in RA patients^30^. In a study on RA fibroblast-like synoviocytes, under inflammatory conditions, an activation of choline metabolism and alteration of phospholipid metabolism have been suggested^68^. Choline is considered essential for the biosynthesis of PL^69^, therefore an increased PL turnover in neurodegenerative diseases like Alzheimer’s disease has been postulated responsible for the decrease of choline concentration^70^.

Notably, the highest concentrations of choline and its metabolite, betaine, were found in RA patients with severe periodontitis or CAL > 5 mm. Circulating levels of high choline and low betaine concentrations have been associated with an unfavourable cardiovascular risk factor profile in healthy individuals from the Hordaland Health Study (HUSK) in Norway^71^. The HUSK study also found a positive relationship between choline and TG, and BMI. We could not find any relationship between choline and TG, or BMI (results not shown) but found a robust negative relationship between choline and HDL-C. HDL-C was found at a lower concentration in RA patients with CAL > 5 mm compared to lower CAL. Of note, in a more recent study on cerebrovascular disease in older adults, higher plasma choline concentrations were associated with a favourable cardiometabolic risk-factor profile, like elevated HDL-concentration^72^.

Choline, or more specifically the microbial metabolism of phosphatidylcholine, has been given much attention due to the production of the pro-atherosclerotic metabolite TMAO (Fig. 1a)^73^. Additionally, l-carnitine serve as precursor for the generation of TMAO (Fig. 1a)^40^. TML and GBB are also known as biosynthetic intermediates in l-carnitine synthesis^74^. In our study, choline, betaine and GBB correlated significantly with TMAO. In humans, TMAO is known to accumulate through the consumption of TMAO-containing seafood^75^. The TMAO concentration in our study correlated positively to whole blood omega-3 index. Recently published data from our group of RA patients revealed a positive relation between marine omega-3 intake and whole blood omega-3 index^43^. Grouping RA patients into TMAO percentiles, the l-carnitine intermediates TML and GBB were elevated in the highest TMAO percentile. CRP and ESR were higher in the second TMAO percentile but did not reach level of significance. Despite differences in several serum carnitine- and choline derivative, TMAO was not related to RA or periodontal disease related variables. Research data covering the causal link between TMAO and other inflammatory diseases like CVD are contradicting^76^. The results of our study seem to reflect the conflicting findings of other studies and further research is needed to establish the role of TMAO in health and disease in humans.

Variables that may potentially affect data interpretation such as age, gender, BMI, smoking status, RA onset, RA duration, periodontal measurements and diagnosis were analysed post recruitment and no statistically significant relations to RA disease activity were found.

However, age, gender, BMI, but also diet- and physical activity dependent differences in FA and lipid composition are well documented findings^77,78^, which should be taken into consideration for the generalizability of the results. RA patients of this study were recruited at a university hospital based rheumatologic outpatient clinic. The overall RA disease activity score in this study measured by DAS28 was at the lower range and may reflect an effective treat-to-target treatment regime with good access to synthetic and biologic disease modifying anti-rheumatic drugs (respectively s-DMARDs and b-DMARDs). Another point of discussion could be the patient-related perceived ability to pass through the examinations that may have influenced the patient's decision to participate in the study, and finally resulted in refusal by patients with more severe RA. Furthermore, using DAS28 score to define RA disease remission means allowing for some degree of residual disease activity. This is also true in this study, as patients with RA in remission showed CRP and ESR levels exceeding the physiological range. Choosing remission cutoff point < 2.6 would reduce, but not exclude, the number of patients with residual disease activity^79^. A DAS28-based definition of remission may therefore be a shortcoming, and more recent guidance documents for RA clinical trials recommendation of the European Medicines Agency and the FDA refer to DAS28 < 2.6 as ‘low disease activity’^80^. Emphasizing awareness, DAS28 score is a well-established and validated tool, especially in clinical studies.

RA treatment with a combination of s-DMARDs and b-DMARDs is currently medical standard. Therefore, limiting patient recruitment to one group of DMARDs is not feasible as it will result in a non-representative patient selection. Furthermore, no differences between active RA and RA in remission were found when grouping RA medication by no, s- and/or b-DMARDs^43^. Prednisolone was significantly related to active RA, but not to periodontal measurements. Glucocorticoids like prednisolone are efficiently used in RA treatment due to their anti-inflammatory and immunosuppressive effects but are also associated with adverse effects depending on several factors like mean and cumulative dose, route of administration, and patient-specific factors^81^. However, adverse effects to low-dose glucocorticoids, as administrated in most RA patients of this study, are less frequent^82^.

Regarding the common effect of smoking on periodontitis, we reported previously higher mean PD (2.9 ± 0.4 mm) and mean CAL (3.2 ± 0.9 mm) in ever smokers compared to never smokers (PD 2.6 ± 0.2 mm, P = 0.002 and CAL 2.8 ± 0.3 mm, *P* = 0.015). Males categorized as ever smokers were found to have the highest mean PD (3.2 ± 0.6 mm) and CAL (4.0 ± 1.4 mm). Further, male sex and smoking were associated with fewer teeth (*P* < 0.001), and more dental plaque (*P* = 0.017) compared to female sex in ever smokers. Between former and current smokers, no differences were found^43^.

In this study, the observed metabolic alterations were related to RA disease activity and prednisolone medication that both were further related to increased CRP. Different degrees of periodontal inflammation in this group of RA patients could not be related to CRP. It can be speculated that the additional inflammatory burden caused by periodontal disease is not able to increase the already elevated inflammatory state caused by RA. Furthermore, RA patients of this study were recruited from urban catchment with good access to dental care (results not shown), and oral health is found improving continuously over the last decades^83^.

The findings of this study should be interpreted with caution within the limits for generalizability and study conceptualization. The influence of periodontal inflammation on the observed metabolic variations was limited, but not absent. Nevertheless, our findings may support the need for interdisciplinary collaboration within the field of medicine and dentistry in patient stratification to improve personalized treatment. Longitudinal studies are warranted to further assess the potential impact of mitochondrial function on RA and periodontitis.

## Supplementary Information

Supplementary Tables.
