# Supplementary material for: Lipid, fatty acid, carnitine- and choline derivative profiles in rheumatoid arthritis outpatients with different degrees of periodontal inflammation
Source: Sci Rep. 2021 Mar 5;11:5332. doi: 10.1038/s41598-021-84122-y (PMC7935865; doi:10.1038/s41598-021-84122-y)
Supplement: Supplementary file 1 — Supplementary Tables. [file 41598_2021_84122_MOESM1_ESM.pdf]

Lipid, fatty acid, carnitine- and choline derivative profiles in rheumatoid arthritis outpatients with different degrees of periodontal inflammation

Kathrin Beyer[1], Stein Atle Lie[1], Bodil Bjørndal[2,3], Rolf K. Berge[2,4], Asbjørn Svardal[2], Johan G. Brun[2,5], Anne Isine Bolstad[1]

1 Department of Clinical Dentistry, Faculty of Medicine, University of Bergen, Bergen, Norway.
2 Department of Clinical Science, University of Bergen, Bergen, Norway.
3 Department of Sport, Food and Natural Sciences, Western Norway University of Applied Sciences, Bergen, Norway.
4 Department of Heart Disease, Haukeland University Hospital, Bergen, Norway
5 Department of Rheumatology, Haukeland University Hospital, Bergen, Norway.

Address correspondence to:

Kathrin Beyer, Department of Clinical Dentistry, University of Bergen, Årstadveien 19, N-5009 Bergen, Norway. E-mail: kathrin.beyer@uib.no. Phone: +47 55 58 65 56.

(Copy to Anne Isine Bolstad, Department of Clinical Dentistry, University of Bergen, Årstadveien 19, N-5009 Bergen, Norway. E-mail: anne.bolstad@.uib.no. Phone: +47 55 58 66 46.)

Supplementary Table S1. Distribution and absolute concentration of whole blood fatty acids (FA) in rheumatoid arthritis (RA) patients (*N* = 78).

	Median	Range	*N* (%) above LOQ
Total FA	3.09	2.12 - 5.45	n.a.
Unidentified FA	0.09	0.04 - 0.12	78 (100)
C14:0, myristic acid	0.02	0.01 - 0.08	78 (100)
C15:0, pentadecylic acid	0.01	0.005 - 0.01	53 (68)
C16:0, palmitic acid	0.73	0.47 - 1.4	78 (100)
C16:1	0.05	0.02 - 0.19	78 (100)
C16:3n-3, HTA	0.01	0.01 - 0.02	78 (100)
C17:0, margaric acid	0.1	0.005 - 0.02	71 (91)
C18:0, stearic acid	0.37	0.27 - 0.52	78 (100)
C18:1	0.55	0.3 - 1.47	78 (100)
C18:2n-6, LA	0.59	0.35 - 0.95	78 (100)
C18:3n-3, ALA	0.02	0.05 - 0.05	78 (100)
C18:4n-3, SDA	0.01	0.05 - 0.1	42 (54)
C20:0, arachidic acid	0.01	0.005 - 0.02	67 (86)
C20:2n-6, eicosadienoic acid	0.01	0.05 - 0.02	76 (97)
C20:3n-6, DGLA	0.04	0.01 - 0.08	78 (100)
C20:4n-6, AA	0.28	0.18 - 0.41	78 (100)
C20:4n-3, ETA	0.005	0.005 - 0.01	3 (4)
C20:5n-3, EPA	0.04	0.01 - 0.17	78 (100)
C22:0, behenic acid	0.01	0.01 - 0.02	78 (100)
C22:4n-6, AdA	0.02	0.005 - 0.05	76 (97)
C22:5n-6, osbond acid	0.005	0.005 - 0.01	10 (8)
C22:5n-3, DPA	0.04	0.03 - 0.05	78 (100)
C22:6n-3, DHA	0.13	0.07 - 0.25	78 (100)

C24:1n-9, nervonic acid	0.02	0.01 - 0.04	78 (100)

Data represent median and range of FA concentration (mg/g), and N, number (%, percentage) of patients with measured FA over limit of quantification (LOQ) <0.01 mg/g. FA below LOQ: C6:0, C8:0, C10:0, C12:0, C16:2n-4, C16:4n-3, C18:3n-6, C20:3n-9, C20:3n-3, C22:1, C21:5n-3, C24:0, C24:5n-5, C24-6n-3. N.a., not applicable; HTA, hexadecatrienoic, LA, linoleic acid; ALA, alpha-linolenic acid; SDA, stearidonic acid; DGLA, dihomo-gamma-linolenic acid; AA, arachidonic acid; ETA, eicosatetraenoic acid; EPA, eicosapentaenoic acid; AdA, adrenic acid; DPA, docosapentaenoic acid; DHA, docosahexaenoic acid.

Supplementary Table S2. Distribution and concentration of whole blood fatty acids (FA), serum lipids and metabolites in rheumatoid arthritis (RA) patients (*N* = 78) grouped by disease activity.

	Total (*N* = 78)	Remission (*N* = 33)	Active RA (*N* = 45)	*P*-value
Total FA (mg)	3.12 ± 0.54	2.98 ± 0.47	3.22 ± 0.57	0.047
SFA (wt%)	37.55 ± 1.95	37.93 ± 2.09	37.28 ± 1.81	0.15
MUFA (wt%)	20.59 ± 2.60	20.11 ± 2.10	20.93 ± 2.89	0.17
PUFA (wt%)	39.16 ± 2.46	39.17 ± 1.96	39.15 ± 2.79	0.99
Unidentified FA (wt%)	2.70 ± 0.64	2.80 ± 0.64	2.64 ± 0.63	0.28
n-3 PUFA (wt%)	8.49 ± 2.25	8.48 ± 2.05	8.50 ± 2.41	0.96
n-6 PUFA (wt%)	30.67 ± 2.49	30.68 ± 2.53	30.65 ± 2.49	0.94
n-3/n-6	0.28 ± 0.09	0.28 ± 0.09	0.28 ± 0.09	0.98
Omega-3 index	6.19 ± 2.09	6.16 ± 1.97	6.22 ± 2.20	0.91
AA/EPA	7.82 ± 5.04	7.42 ± 4.21	8.11 ± 5.59	0.55
EPA/ALA	3.22 ± 2.29	3.25 ± 2.46	3.19 ± 2.19	0.92
AA/LA	0.50 ± 0.11	0.50 ± 0.11	0.50 ± 0.11	0.90
AIFA index	765.63 ± 195.61	759.88 ± 183.21	769.84 ± 206.17	0.83
DNL index	1.27 ± 0.21	1.28 ± 0.25	1.26 ± 0.19	0.71
Elongase C18:0/C16:0	0.51 ± 0.05	0.52 ± 0.06	0.50 ± 0.05	0.10
Desaturase D5n-6	7.52 ± 3.06	8.05 ± 4.25	7.12 ± 1.71	0.19
C14:0 (wt%)	0.86 ± 0.37	0.82 ± 0.37	0.90 ± 0.37	0.35
C15:0 (wt%)	0.24 ± 0.10	0.21 ± 0.07	0.27 ± 0.10	0.010
C16:0 (wt%)	23.66 ± 1.61	23.75 ± 1.88	23.60 ± 1.41	0.68
C16:1 (wt%)	1.62 ± 0.56	1.54 ± 0.57	1.69 ± 0.55	0.25
C16:3n-3 (wt%)	0.34 ± 0.12	0.31 ± 0.11	0.36 ± 0.11	0.029
C17:0 (wt%)	0.28 ± 0.13	0.31 ± 0.15	0.25 ± 0.11	0.032

C18:0 (wt%)	11.92 ± 0.97	12.21 ± 1.03	11.7 ± 0.88	0.022
C18:1 (wt%)	18.05 ± 2.32	17.67 ± 1.81	18.32 ± 2.62	0.22
C18:2n-6, LA (wt%)	18.94 ± 2.10	18.93 ± 2.31	18.94 ± 1.95	0.99
C18:3n-3, ALA (wt%)	0.61 ± 0.24	0.62 ± 0.25	0.61 ± 0.24	0.94
C18:4n-3 (wt%)	0.13 ± 0.08	0.12 ± 0.09	0.14 ± 0.08	0.36
C20:0 (wt%)	0.24 ± 0.08	0.26 ± 0.09	0.23 ± 0.07	0.038
C20:1 (wt%)	0.18 ± 0.06	0.17 ± 0.06	0.19 ± 0.06	0.17
C20:2n-6 (wt%)	0.33 ± 0.12	0.34 ± 0.17	0.31 ± 0.07	0.30
C20:3n-6, DGLA (wt%)	1.33 ± 0.33	1.30 ± 0.36	1.35 ± 0.31	0.46
C20:4n-6, AA (wt%)	9.25 ± 1.43	9.25 ± 1.30	9.26 ± 1.54	0.98
C20:4n-3 (wt%)	0.01 ± 0.05	0.01 ± 0.05	0.01 ± 0.05	0.91
C20:5n-3, EPA (wt%)	1.74 ± 1.13	1.74 ± 1.21	1.74 ± 1.08	1
C22:0 (wt%)	0.35 ± 0.07	0.37 ± 0.07	0.35 ± 0.07	0.20
C22:4n-6 (wt%)	0.79 ± 0.29	0.85 ± 0.31	0.75 ± 0.28	0.15
C22:5n-6 (wt%)	0.03 ± 0.09	0.02 ± 0.07	0.03 ± 0.10	0.59
C22:5n-3, DPA (wt%)	1.21 ± 0.22	1.26 ± 0.21	1.16 ± 0.21	0.049
C22:6n-3, DHA (wt%)	4.46 ± 1.08	4.43 ± 0.89	4.48 ± 1.21	0.83
C24:1n-9 (wt%)	0.73 ± 0.16	0.73 ± 0.16	0.73 ± 0.17	0.96
Lipids				
TG (mmol/L)	1.20 ± 0.82	1.12 ± 0.65	1.26 ± 0.92	0.43
TC (mmol/L)	5.52 ± 1.22	5.33 ± 1.02	5.64 ± 1.34	0.26
HDL-C (mmol/L)	1.64 ± 0.50	1.57 ± 0.44	1.69 ± 0.53	0.30
LDL-C (mmol/L)	3.29 ± 0.93	3.24 ± 0.76	3.33 ± 1.05	0.69
PL (mmol/L)	3.04 ±0.55	2.94 ± 0.48	3.11 ± 0.60	0.19
NEFA (mmol/L)	0.51 ±0.27	0.47 ± 0.21	0.53 ± 0.30	0.31
HDL-C/LDL-C ratio	0.54 ± 0.24	0.51 ± 0.19	0.56 ± 0.27	0.37
Non HDL-C (mmol/L)	3.87 ± 1.15	3.76 ± 0.89	3.95 ± 1.32	0.46

Castelli's risk index	3.63 ± 1.36	3.55 ± 0.86	3.70 ± 1.63	0.63
Atherogenic index	-0.17 ± 0.30	-0.18 ± 0.27	-0.17 ± 0.33	0.91
Metabolites				
Betaine (µM)	40.32 ± 19.30	38.01 ± 14.03	42.01 ± 22.41	0.37
Choline (µM)	10.73 ± 2.46	10.60 ± 2.67	10.83 ± 2.32	0.68
L-carnitine (µM)	36.66 ± 8.07	37.28 ± 9.51	36.21 ± 6.90	0.57
GBB (µM)	0.91 ± 0.22	0.92 ± 0.25	0.89 ± 0.19	0.53
TML (µM)	0.49 ± 0.18	0.45 ± 0.13	0.52 ± 0.20	0.10
TMAO (µM)	6.57 ± 7.91	6.19 ± 6.72	6.86 ± 8.74	0.71
Acetyl-C, AC2 (µM)	7.26 ± 2.82	7.09 ± 2.45	7.38 ± 3.09	0.66
Propionyl-C, AC3 (µM)	0.37 ± 0.11	0.37 ± 0.12	0.37 ± 0.10	0.95
Valeryl-C, AC5 (µM)	0.17 ± 0.49	0.24 ± 0.75	0.12 ± 0.05	0.28
Octanoyl-C, AC8 (µM)	0.14 ± 0.06	0.12 ± 0.04	0.15 ± 0.06	0.043
Lauroyl-C, AC12 (µM)	0.06 ± 0.03	0.05 ± 0.02	0.06 ± 0.03	0.034
Myristoyl-C, AC14 (µM)	0.03 ± 0.01	0.02 ± 0.01	0.03 ± 0.01	0.10
Palmitoyl-C, AC16 (µM)	0.11 ± 0.03	0.10 ± 0.03	0.11 ± 0.03	0.55
AC2 /AC8	57.76 ± 20.12	61.91 ± 18.39	54.72 ± 20.97	0.12
AC2 /AC12	138.56 ± 53.12	148.54 ± 43.04	131.23 ± 58.83	0.16
AC2 /AC14	283.65 ± 87.17	302.08 ± 90.28	270.14 ± 83.23	0.11
AC2 /AC16	69.38 ± 19.93	69.80 ± 20.14	69.08 ± 20.00	0.88
AC3 /AC16	3.74 ± 1.35	3.81 ± 1.52	3.69 ± 1.23	0.69

Data represent mean ± SD, standard deviation of observed values. RA disease activity, measured as disease activity score 28 (DAS28); Remission, RA disease in remission defined as DAS28≤2.6; Active RA disease defined as DAS28>2.6; N, number; SFA, saturated FA; MUFA, monounsaturated FA; PUFA, polyunsaturated FA; n-3 PUFA, sum of n-3 PUFA; n-6 PUFA, sum of n-6 PUFA; Omega-3 index, sum of wt% EPA, eicosapentaenoic acid, and wt% DHA, docosahexaenoic acid; AA, arachidonic acid; ALA, alpha-linolenic acid; LA, linoleic acid; AIFA index, anti-inflammatory fatty acid index; DNL index, de novo lipogenesis index; DGLA, dihomo-gamma-linolenic acid; DPA, docosapentaenoic acid; TG, triglyceride; TC, total cholesterol; HDL-C, high-density lipoprotein cholesterol; LDL-C, low-density lipoprotein

cholesterol; PL, phospholipid; NEFA, non-esterified FA; Castelli`s risk index, ratio of TC to HDL-C; Atherogenic index, log10(TG/HDL-C); GBB, γ-butyrobetaine; TML, trimethyl lysine; TMAO, trimethylamine N-oxide; Acetyl-C (AC2), Acetyl-Carnitine. Analysed with Student t-test: *P*-level <0.05.

Supplementary Table S3. Rheumatoid arthritis patient characteristics grouped by prednisolone medication. Categorical/continuous variables of demographic, behavioral and clinical characteristics, and distribution and concentration of whole blood fatty acids (FA), serum lipids and metabolites.

	Total (*N* = 78)	No prednisolone (*N* = 57)	Prednisolone use (*N* = 21)	*P*-value
Females (N/%)	57/73	18/32	39/68	0.13
Age (years)	57 ± 12	57 ± 12	57 ± 11	0.95
BMI (kg/m^2)	26.1 ± 4.3	26.4 ± 4.5	25.4 ± 4.1	0.40
Smoking status (N/%)				
Never smokers	29/37	18/32	11/52	
Former smokers	35/45	28/49	7/33	0.24
Current smokers	14/19	11/19	7/14	
Oral data				
Number of teeth	25 ± 5	25 ± 5	24 ± 6	0.53
PD (mm)	2.8 ± 0.4	2.7 ± 0.4	2.8 ± 0.5	0.41
CAL (mm)	3.1 ± 0.8	3.0 ± 0.7	3.1 ± 1.0	0.50
BoP (% of sites)	31 ± 16	32 ± 5	28 ± 16	0.26
PI (% of sites)	33 ± 16	35 ± 17	27 ± 12	0.05
Periodontitis (N/%)				
No	14/18	9/16	5/24	
Mild/ moderate	50/64	39/68	11/52	0.24
Severe	14/18	9/16	5/24	
RA data				
Active RA (N/%)	45/58	28/49	17/81	0.012
RA debut (age)	43 ± 14	42 ± 14	45 ± 16	0.51
RA duration (years)	15 ± 11	15 ± 12	14 ± 11	0.65

DAS28 (score)	3.0 ± 1.1	2.7 ± 0.9	3.7 ± 1.3	0.001
VAS (score)	28 ± 21	25 ± 18	35 ± 25	0.07
MHAQ (score)	0.37 ± 0.38	0.34 ± 0.37	0.44 ±0.40	0.30
RF, ACPA (N/%)	60/77	45/58	15/19	0.48
Laboratory data				
ESR (mm/hr)	19.8 ± 15.5	16.7 ± 12.3	28.3 ± 20.1	0.003
CRP (mg/L)	8.8 ± 13.5	6.5 ± 10.1	15.1 ±18.9	0.012
RF (IU/ml) †	184 ± 385	202 ± 421	132 ± 270	0.49
FA data				
Total FA (mg)	3.12 ± 0.54	3.05 ± 0.58	3.32 ± 0.37	0.043
SFA (wt%)	37.55 ± 1.95	37.53 ± 1.95	37.63 ± 1.99	0.84
MUFA (wt%)	20.59 ± 2.60	20.47 ± 2.78	20.89 ± 2.06	0.54
PUFA (wt%)	39.16 ± 2.46	39.18 ± 2.42	39.10 ± 2.62	0.91
Unidentified FA (wt%)	2.70 ± 0.64	2.82 ± 0.63	2.83 ± 0.54	0.006
n-3 PUFA (wt%)	8.49 ± 2.25	8.44 ± 2.34	8.62 ± 2.02	0.76
n-6 PUFA (wt%)	30.67 ± 2.49	30.74 ± 2.49	30.48 ± 2.54	0.69
n-3/n-6	0.28 ± 0.09	0.28 ± 0.10	0.29 ± 0.08	0.77
Omega-3 index	6.19 ± 2.09	6.18 ± 5.60	6.24 ± 5.38	0.91
AA/EPA	7.82 ± 5.04	8.26 ± 5.11	6.63 ± 4.73	0.21
EPA/ALA	3.22 ± 2.29	3.29 ± 2.49	3.03 ± 1.70	0.67
AA/LA	0.50 ± 0.11	0.51 ± 0.11	0.47 ± 0.10	0.25
AIFA index	765.63 ± 195.61	764.22 ± 202.90	769.45 ± 178.95	0.92
DNL index	1.27 ± 0.21	1.27 ± 0.21	1.27 ± 1.17	0.97
Elongase C18:0/C16:0	0.51 ± 0.05	0.52 ± 0.05	0.48 ± 0.04	0.013
Desaturase D5n-6	7.52 ± 3.06	7.69 ± 3.45	7.03 ± 1.59	0.40
C14:0 (wt%)	0.86 ± 0.37	0.79 ± 0.30	1.07 ± 0.46	0.002
C15:0 (wt%)	0.24 ± 0.10	0.24 ± 0.22	0.25 ± 0.12	0.61

C16:0 (wt%)	23.66 ± 1.61	23.56 ± 23.12	23.93 ± 1.45	0.37
C16:1 (wt%)	1.62 ± 0.56	1.60 ± 0.58	1.69 ± 0.49	0.53
C16:3n-3 (wt%)	0.34 ± 0.12	0.33 ± 0.12	0.36 ± 0.11	0.38
C17:0 (wt%)	0.28 ± 0.13	0.28 ± 0.13	0.28 ± 0.23	0.90
C18:0 (wt%)	11.92 ± 0.97	12.07 ± 1.0	11.51 ± 0.78	0.023
C18:1 (wt%)	18.05 ± 2.32	17.95 ± 2.50	18.29 ± 1.77	0.57
C18:2n-6, LA (wt%)	18.94 ± 2.10	18.85 ± 2.15	19.17 ± 1.98	0.56
C18:3n-3, ALA (wt%)	0.61 ± 0.24	0.58 ± 0.23	0.70 ± 0.24	0.06
C18:4n-3 (wt%)	0.13 ± 0.08	0.12 ± 0.08	0.17 ± 0.07	0.011
C20:0 (wt%)	0.24 ± 0.08	0.24 ± 0.08	0.24 ± 0.09	0.91
C20:1 (wt%)	0.18 ± 0.06	0.18 ± 0.06	0.19 ± 0.06	0.76
C20:2n-6 (wt%)	0.33 ± 0.12	0.32 ± 0.14	0.32 ± 0.06	0.77
C20:3n-6, DGLA (wt%)	1.33 ± 0.33	1.33 ± 0.34	1.32 ± 0.29	0.91
C20:4n-6, AA (wt%)	9.25 ± 1.43	9.36 ± 1.39	8.79 ± 1.54	0.29
C20:4n-3 (wt%)	0.01 ± 0.05	0.01 ± 0.05	0.01 ± 0.04	0.82
C20:5n-3, EPA (wt%)	1.74 ± 1.13	1.67 ± 1.18	1.91 ± 0.99	0.42
C22:0 (wt%)	0.35 ± 0.07	0.36 ± 0.07	0.34 ± 0.07	0.35
C22:4n-6, AdA (wt%)	0.79 ± 0.29	0.83 ± 0.28	0.68 ± 0.30	0.031
C22:5n-6 (wt%)	0.03 ± 0.09	0.03 ± 0.09	0.03 ± 0.07	1.0
C22:5n-3, DPA (wt%)	1.21 ± 0.22	1.22 ± 0.23	1.15 ± 0.14	0.18
C22:6n-3, DHA (wt%)	4.46 ± 1.08	4.51 ± 1.11	4.33 ± 0.99	0.53
C24:1n-9 (wt%)	0.73 ± 0.16	0.74 ± 0.16	0.72 ± 0.64	0.62
Lipids				
TG (mmol/L)	1.20 ± 0.82	1.17 ± 0.90	1.29± 0.55	0.58
TC (mmol/L)	5.52 ± 1.22	5.35 ± 1.19	5.95 ± 2.21	0.05
HDL-C (mmol/L)	1.64 ± 0.50	1.58 ± 0.45	1.81 ± 0.57	0.06
LDL-C (mmol/L)	3.29 ± 0.93	3.21 ± 0.90	3.52 ± 1.00	0.19

PL (mmol/L)	3.04 ±0.55	2.95 ± 0.54	3.29 ± 0.51	0.015
NEFA (mmol/L)	0.51 ±0.27	0.51 ± 0.21	0.50 ± 0.40	0.89
HDL-C/LDL-C ratio	0.54 ± 0.24	0.54 ± 0.23	0.55 ± 0.25	0.80
Non HDL-C (mmol/L)	3.87 ± 1.15	3.77 ± 1.17	4.14 ± 1.08	0.22
Castelli's risk index	3.63 ± 1.36	3.68 ± 1.48	3.51 ± 0.97	0.62
Atherogenic index	-0.17 ± 0.30	-0.18 ± 0.32	-0.16 ± 0.27	0.81
Metabolites				
Betaine (µM)	40.32 ± 19.30	42.21 ± 21.41	35.18 ± 10.65	0.16
Choline (µM)	10.73 ± 2.46	11.12 ± 2.43	9.69 ± 2.29	0.022
L-carnitine (µM)	36.66 ± 8.07	37.82 ± 8.05	33.53 ± 7.42	0.036
GBB (µM)	0.91 ± 0.22	0.95 ± 0.22	0.79 ± 0.17	0.003
TML (µM)	0.49 ± 0.18	0.50 ± 0.19	0.47 ± 0.13	0.47
TMAO (µM)	6.57 ± 7.91	6.18± 7.60	7.63 ± 8.80	0.48
Acetyl-C, AC2 (µM)	7.26 ± 2.82	7.75 ± 2.83	5.92 ± 2.36	0.010
Propionyl-C, AC3 (µM)	0.37 ± 0.11	0.38 ± 0.12	0.35 ± 0.09	0.38
Valeryl-C, AC5 (µM)	0.17 ± 0.49	0.19 ± 0.57	0.13 ± 0.06	0.64
Octanoyl-C, AC8 (µM)	0.14 ± 0.06	0.14 ± 0.05	0.13 ± 0.06	0.43
Lauroyl-C, AC12 (µM)	0.06 ± 0.03	0.06 ± 0.03	0.06 ± 0.03	0.56
Myristoyl-C, AC14 (µM)	0.03 ± 0.01	0.03 ± 0.01	0.03 ± 0.01	0.39
Palmitoyl-C, AC16 (µM)	0.11 ± 0.03	0.11 ± 0.03	0.09 ± 0.02	0.05
AC2 /AC8	57.76 ± 20.12	60.16 ± 20.50	51.25 ± 17.89	0.08
AC2 /AC12	138.56 ± 53.12	146.18 ± 54.87	117.92 ± 42.68	0.036
AC2 /AC14	283.65 ± 87.17	299.47 ± 89.47	240.71 ± 64.73	0.007
AC2 /AC16	69.38 ± 19.93	71.99 ± 20.07	62.30 ± 18.13	0.06
AC3 /AC16	3.74 ± 1.35	3.66 ± 1.36	3.08 ± 1.33	0.39

Data represent mean ± SD, standard deviation of observed values. N/%, number/percentage; Age, patient`s age in years at the time of clinical examination; BMI, body mass index; PD, probing depth; CAL, clinical attachment level; BoP, bleeding on probing; PI, plaque index; RA disease

activity, measured as disease activity score 28 (DAS28); Remission, RA disease in remission defined as DAS28≤2.6; Active RA disease defined as DAS28>2.6; VAS, patient global health assessment score on a visual analogue scale; MHAQ, modified health assessment questionnaire; RF, ACPA, seropositivity, positive tests for rheumatoid factor (RF) (N=74) and/or anti-citrullinated protein antibody (ACPA) (N=77); ESR, erythrocyte sedimentation rate; CRP, C-reactive protein. SFA, saturated FA; MUFA, monounsaturated FA; PUFA, polyunsaturated FA; n-3 PUFA, sum of n-3 PUFA; n-6 PUFA, sum of n-6 PUFA; Omega-3 index, sum of wt% EPA, eicosapentaenoic acid, and wt% DHA, docosahexaenoic acid; AA, arachidonic acid; ALA, alpha-linolenic acid; LA, linoleic acid; AIFA index, anti-inflammatory fatty acid index; DNL index, de novo lipogenesis index; DGLA, dihomo-gamma-linolenic acid; AdA, adrenic acid; DPA, docosapentaenoic acid; TG, triglyceride; TC, total cholesterol; HDL-C, high-density lipoprotein cholesterol; LDL-C, low-density lipoprotein cholesterol; PL, phospholipid; NEFA, non-esterified FA; Castelli`s risk index, ratio of TC to HDL-C; Atherogenic index, log10(TG/HDL-C); GBB, γ-butyrobetaine; TML, trimethyl lysine; TMAO, trimethylamine N-oxide; Acetyl-C (AC2), Acetyl-Carnitine. Analysed with Pearson chi-square and Student t-test: P-level <0.05. † n = 74

Supplementary Table S4. Distribution of demographic, behavioral and clinical continuous variables including lipid-, metabolite- and fatty acid (FA) concentrations in rheumatoid arthritis (RA) patients (*N* = 78) grouped by periodontal diagnosis.

	Periodontitis			
Variables	No (*N* = 14)	Mild/ moderate (*N* = 50)	Severe (*N* = 14)	*P*-value
Age (years)	54 ± 11	56 ± 12	63 ± 9	0.07
BMI (kg/m^2)	27 ± 4	25 ± 4	28 ± 5	0.10
Oral data				
Number of teeth	27 ± 2	25 ± 5	22 ± 6	0.016
PD (mm)	2.4 ± 0.1	2.7 ± 0.2	3.3 ± 0.5	<0.001
CAL (mm)	2.6 ± 0.2	2.9 ± 0.4	4.1 ± 1.2	<0.001
BoP (%)	22 ± 11	29 ± 13	47 ± 17	<0.001
PI (%)	24 ± 9	33 ± 15	42 ± 22	0.016
RA data				
RA debut (age)	40 ± 13	41 ± 15	53 ± 10	0.020
RA duration (years)	15 ± 10	16 ± 12	11 ± 10	0.42
DAS28 (score)	2.8 ± 0.9	3.0 ± 1.2	3.2 ± 1.1	0.75
VAS (score)	29 ± 21	25 ± 19	39 ± 25	0.06
MHAQ (score)	0.31 ± 0.42	0.35 ± 0.36	0.49 ± 0.40	0.40
Laboratory data				
RF (IU/ml) [†]	162 ± 496	217 ± 402	96 ± 119	0.58
CRP (mg/L)	5 ± 5	9 ± 15	13 ± 15	0.35
ESR (mm/hr)	17 ± 13	21 ± 17	18 ± 12	0.64
FA				
Total FA (mg)	3.47 ± 0.75	3.02 ± 0.45	3.13 ± 0.46	0.021
SFA (wt%)	37.39 ± 1.60	37.71 ± 2.02	37.18 ± 2.06	0.63
MUFA (wt%)	21.18 ± 3.38	20.17 ± 2.26	21.49 ± 2.75	0.16
PUFA (wt%)	39.09 ± 3.12	39.35 ± 2.45	38.54 ± 1.72	0.55
Unidentified FA (wt%)	2.34 ± 0.65	2.78 ± 0.63	2.79 ± 0.58	0.06
n-3 PUFA (wt%)	8.97 ± 2.95	8.62 ± 2.17	7.58 ± 1.48	0.22
n-6 PUFA (wt%)	30.13 ± 2.66	30.74 ± 2.48	30.96 ± 2.46	0.65
n-3/n-6	0.30 ± 0.12	0.29 ± 0.09	0.25 ± 0.07	0.27
Omega-3 index	6.50 ± 2.85	6.35 ± 2.00	5.33 ± 1.30	0.23
AA/EPA	7.08 ± 5.01	7.70 ± 5.26	8.97 ± 4.32	0.60
EPA/ALA	3.38 ± 3.51	3.38 ± 1.96	2.48 ± 1.91	0.42
AA/LA	0.46 ± 0.11	0.51 ± 0.11	0.48 ± 0.10	0.31
AIFA index	787.11 ± 269.33	779.85 ± 186.64	693.38 ± 126.14	0.31
DNL index	1.26 ± 0.20	1.28 ± 0.23	1.24 ± 0.18	0.77
Elongase C18:0/C16:0	0.49 ± 0.06	0.51 ± 0.05	0.50 ± 0.05	0.21
Desaturase D5n-6	7.59 ± 2.66	7.80 ± 3.43	6.41 ± 1.57	0.33
C14:0 (wt%)	1.01 ± 0.35	0.83 ± 0.37	0.85 ± 0.36	0.28
C15:0 (wt%)	0.25 ± 0.12	0.25 ± 0.09	0.23 ± 0.10	0.88

C16:0 (wt%)	23.80 ± 1.33	23.67 ± 1.74	23.46 ± 1.46	0.86
C16:1 (wt%)	1.73 ± 0.72	1.57 ± 0.55	1.73 ± 0.39	0.47
C16:3n-3 (wt%)	0.37 ± 0.13	0.33 ± 0.12	0.33 ± 0.10	0.92
C17:0 (wt%)	0.27 ± 0.11	0.28 ± 0.13	0.28 ± 0.12	0.92
C18:0 (wt%)	11.51 ± 1.18	12.08 ± 0.87	11.77 ± 1.01	0.13
C18:1 (wt%)	18.53 ± 2.91	17.68 ± 2.05	18.85 ± 2.47	0.17
C18:2n-6, LA (wt%)	19.15 ± 2.23	18.81 ± 2.18	19.20 ± 1.71	0.77
C18:3n-3, ALA (wt%)	0.74 ± 0.27	0.56 ± 0.20	0.67 ± 0.31	0.026
C18:4n-3 (wt%)	0.17 ± 0.07	0.13 ± 0.08	0.12 ± 0.09	0.18
C20:0 (wt%)	0.24 ± 0.06	0.24 ± 0.09	0.24 ± 0.08	0.99
C20:1 (wt%)	0.19 ± 0.07	0.18 ± 0.06	0.18 ± 0.05	0.91
C20:2n-6 (wt%)	0.30 ± 0.08	0.35 ± 0.13	0.30 ± 0.12	0.48
C20:3n-6, DGLA (wt%)	1.24 ± 0.32	1.32 ± 0.33	1.48 ± 0.29	0.14
C20:4n-6, AA (wt%)	8.74 ± 1.56	9.42 ± 1.32	9.16 ± 1.68	0.28
C20:4n-3 (wt%)	0.03 ± 0.08	0.01 ± 0.04	0 ± 0	0.16
C20:5n-3, EPA (wt%)	2.08 ± 1.68	1.78 ± 1.03	1.24 ± 0.62	0.14
C22:0 (wt%)	0.33 ± 0.07	0.36 ± 0.08	0.35 ±	0.28
C22:4n-6, Ada (wt%)	0.70 ± 0.31	0.82 ± 0.27	0.79 ± 0.33	0.40
C22:5n-6 (wt%)	0 ± 0	0.03 ± 0.10	0.03 ± 0.07	0.44
C22:5n-3, DPA (wt%)	0.16 ± 0.28	1.24 ± 0.21	1.13 ± 0.15	0.15
C22:6n-3, DHA (wt%)	4.42 ± 1.28	4.57 ± 1.07	4.09 ± 0.85	0.33
C24:1n-9 (wt%)	0.73 ± 0.20	0.73 ± 0.17	0.73 ± 0.13	1
Lipids				
TG (mmol/L)	1.68 ± 1.40	1.01 ± 0.44	1.42 ± 0.92	0.013
TC (mmol/L)	5.78 ± 1.67	5.38 ± 1.05	5.69 ± 1.30	0.47
HDL-C (mmol/L)	1.73 ± 0.43	1.69 ± 0.53	1.38 ± 0.36	0.09
LDL-C (mmol/L)	3.27 ± 1.06	2.21 ± 0.85	3.58 ± 1.09	0.44
PL (mmol/L)	3.32 ± 0.74	2.96 ± 0.47	3.06 ± 0.57	0.10
NEFA (mmol/L)	0.55 ± 0.24	0.50 ± 0.30	0.48 ± 0.18	0.79
HDL-C/LDL-C	0.58 ± 0.21	0.57 ± 0.26	0.42 ± 0.14	0.09
Non HDL-C (mmol/L)	4.05 ± 1.59	3.70 ± 0.98	4.31 ± 1.18	0.17
Castelli's risk index	3.47 ± 1.12	3.5 ± 1.44	4.27 ± 1.14	0.15
Atherogenic index	-0.09 ± 0.32	-0.24 ± 0.29	-0.04 ± 0.30	0.049
Metabolites				
Betaine (µM)	33.52 ± 7.34	38.97 ± 12.79	51.94 ± 36.54	0.027
Choline (µM)	9.74 ± 1.39	10.60 ± 2.28	12.20 ± 3.31	0.023
L-carnitine (µM)	36.60 ± 6.75	35.43 ± 8.54	41.13 ± 6.14	0.06
GBB (µM)	0.85 ± 0.18	0.91 ± 0.22	0.96 ± 0.24	0.44
TML (µM)	0.45 ± 0.13	0.48 ± 0.16	0.59 ± 0.24	0.06
TMAO (µM)	4.57 ± 3.01	7.46 ± 9.58	5.42 ± 2.51	0.41
Acetyl-C, AC2 (µM)	7.24 ± 2.67	7.31 ± 3.08	7.07 ± 2.02	0.96
Propionyl-C, AC3 (µM)	0.39 ± 0.14	0.35 ± 0.09	0.44 ± 0.12	0.015
Valeryl-C, AC5 (µM)	0.13 ± 0.08	0.19 ± 0.61	0.13 ± 0.04	0.88
Octanoyl-C, AC8 (µM)	0.14 ± 0.06	0.13 ± 0.06	0.15 ± 0.06	0.57
Lauroyl-C, AC12 (µM)	0.06 ± 0.03	0.06 ± 0.03	0.07 ± 0.04	0.46
Myristoyl-C, AC14 (µM)	0.03 ± 0.01	0.03 ± 0.01	0.03 ± 0.02	0.25

Palmitoyl-C, AC16 (µM)	0.11 ± 0.04	0.10 ± 0.03	0.11 ± 0.03	0.41
AC2 /AC8	56.49 ± 21.47	60.25 ± 21.20	50.14 ± 12.40	0.25
AC2 /AC12	136.61 ± 53.85	144.67 ± 57.18	118.66 ± 30.28	0.27
AC2 /AC14	273.91 ± 65.73	296.02 ± 95.09	249.24 ± 68.11	0.19
AC2 /AC16	69.08 ± 19.20	71.04 ± 21.01	63.79 ± 16.58	0.49
AC3 /AC16	3.80 ± 1.31	3.63 ± 1.35	4.08 ± 1.43	0.54

Data represent mean ± SD, standard deviation of observed values. Age, age in years at the time of clinical examination; BMI, body mass index; PD, probing depth; CAL, clinical attachment level; BoP, percentage of sites with bleeding on probing; PI, percentage of sites with dental plaque; RA debut, age when diagnosed with RA; DAS28, RA disease activity score; VAS, patient global health assessment score on a visual analogue scale; MHAQ, modified health assessment questionnaire; RF, rheumatoid factor; CRP, C-reactive protein; ESR, erythrocyte sedimentation rate; SFA, saturated FA; MUFA, monounsaturated FA; PUFA, polyunsaturated FA; n-3 PUFA, sum of n-3 PUFA; n-6 PUFA, sum of n-6 PUFA; Omega-3 index, sum of wt% EPA, eicosapentaenoic acid, and wt% DHA, docosahexaenoic acid; AA, arachidonic acid; ALA, alpha-linolenic acid; LA, linoleic acid; AIFA index, anti-inflammatory fatty acid index; DNL index, de novo lipogenesis index; DGLA, dihomo-gamma-linolenic acid; AdA, adrenic acid; DPA, docosapentaenoic acid; TG, triglyceride; TC, total cholesterol; HDL-C, high-density lipoprotein cholesterol; LDL-C, low-density lipoprotein cholesterol; PL, phospholipid; NEFA, non-esterified FA; Castelli's risk index, ratio TC/HDL-C; Atherogenic index, log10(TG/HDL-C); GBB, γ-butyrobetaine; TML, trimethyl lysine; TMAO, trimethylamine N-oxide; Acetyl-C (AC2), Acetyl-Carnitine. Analysed with one-way ANOVA: *P*-level <0.05. † n = 74

Supplementary Table S5. Distribution of demographic, behavioral and clinical continuous variables including lipid-, metabolite- and fatty acid (FA) concentrations in rheumatoid arthritis (RA) patients (*N* = 78) grouped by clinical attachment level (CAL) ≤ 5mm CAL > 5mm.

Variables	CAL ≤ 5mm (*N* = 50)	CAL > 5mm (*N* = 28)	*P*-value
Age (years)	53 ± 11	64 ± 9	<0.001
BMI (kg/m^2)	26 ± 5	27 ± 4	0.20
Oral data			
Number of teeth	26 ± 4	23 ± 6	0.005
PD (mm)	2.6 ± 0.2	3.0 ± 0.5	<0.001
CAL (mm)	2.7 ± 0.2	3.7 ± 1.0	<0.001
BoP (%)	27 ± 13	39 ± 17	0.001
PI (%)	28 ± 11	42 ± 20	<0.001
RA data			
RA debut (age)	38 ± 13	52 ± 12	<0.001
RA duration (years)	16 ± 12	12 ± 11	0.17
DAS28 (score)	2.9 ± 1.1	3.1 ± 1.1	0.49
VAS (score)	27 ± 19	30 ± 23	0.59
MHAQ (score)	0.35 ± 0.39	0.41 ± 0.37	0.48
Laboratory data			
RF (IU/ml) †	191 ± 437	170 ± 271	0.83
CRP (mg/L)	8 ± 14	10 ± 12	0.55
ESR (mm/hr)	18 ± 16	23 ± 15	0.17
FA data			
Total FA (mg)	3.12 ± 0.60	3.12 ± 0.42	0.96
SFA (wt%)	37.49 ± 1.74	37.67 ± 2.30	0.70
MUFA (wt%)	20.53 ± 2.70	20.68 ± 2.46	0.81
PUFA (wt%)	39.32 ± 2.65	38.88 ± 2.10	0.45
Unidentified FA (wt%)	2.67 ± 0.67	2.77 ± 0.57	0.49
n-3 PUFA (wt%)	8.53 ± 2.41	8.43 ± 1.95	0.86
n-6 PUFA (wt%)	30.79 ± 2.54	30.44 ± 2.43	0.56
n-3/n-6	0.28 ± 0.10	0.28 ± 0.08	0.99
Omega-3 index	6.18 ± 2.27	6.22 ± 1.77	0.93
AA/EPA	8.18 ± 5.40	7.18 ± 4.33	0.41
EPA/ALA	3.01 ± 2.31	3.59 ± 2.72	0.28
AA/LA	0.50 ± 0.11	0.50 ± 0.10	0.84
AIFA index	761.17 ± 211.62	773.60 ± 166.58	0.79
DNL index	1.26 ± 0.20	1.29 ± 0.24	0.56
Elongase C18:0/C16:0	0.51 ± 0.05	0.50 ± 0.05	0.58
Desaturase D5n-6	7.88 ± 3.62	6.82 ± 1.50	0.16
C14:0 (wt%)	0.88 ± 0.39	0.84 ± 0.33	0.68
C15:0 (wt%)	0.24 ± 0.10	0.25 ± 0.08	0.80
C16:0 (wt%)	23.55 ± 1.53	23.85 ± 1.76	0.44
C16:1 (wt%)	1.60 ± 0.59	1.67 ± 0.50	0.56

C16:3n-3 (wt%)	0.35 ± 0.12	0.32 ± 0.11	0.32
C17:0 (wt%)	0.30 ± 0.14	0.25 ± 0.10	0.11
C18:0 (wt%)	11.92 ± 0.89	11.92 ± 1.12	0.98
C18:1 (wt%)	18.03 ± 2.39	18.08 ± 2.23	0.93
C18:2n-6, LA (wt%)	18.99 ± 2.10	18.85 ± 2.13	0.78
C18:3n-3, ALA (wt%)	0.63 ± 0.22	0.59 ± 0.28	0.54
C18:4n-3 (wt%)	0.13 ± 0.08	0.13 ± 0.08	0.82
C20:0 (wt%)	0.25 ± 0.09	0.22 ± 0.06	0.09
C20:1 (wt%)	0.18 ± 0.06	0.18 ± 0.06	1
C20:2n-6 (wt%)	0.34 ± 0.13	0.30 ± 0.10	0.14
C20:3n-6, DGLA (wt%)	1.30 ± 0.34	1.38 ± 0.30	0.29
C20:4n-6, AA (wt%)	9.32 ± 1.49	9.14 ± 1.35	0.59
C20:4n-3 (wt%)	0.02 ± 0.06	0.01 ± 0.02	0.25
C20:5n-3, EPA (wt%)	1.75 ± 1.25	1.71 ± 0.91	0.86
C22:0 (wt%)	0.36 ± 0.07	0.35 ± 0.08	0.50
C22:4n-6, AdA (wt%)	0.83 ± 0.30	0.73 ± 0.28	0.17
C22:5n-6 (wt%)	0.02 ± 0.07	0.05 ± 0.11	0.14
C22:5n-3, DPA (wt%)	1.23 ± 0.23	1.17 ± 0.20	0.26
C22:6n-3, DHA (wt%)	4.43 ± 1.14	4.52 ± 0.99	0.72
C24:1n-9 (wt%)	0.72 ± 0.17	0.75 ± 0.15	0.48
Lipids			
TG (mmol/L)	1.20 ± 0.87	1.20 ± 0.72	0.99
TC (mmol/L)	5.51 ± 1.24	5.51 ± 1.20	0.98
HDL-C (mmol/L)	1.72 ± 0.49	1.49 ± 0.48	0.048
LDL-C (mmol/L)	3.23 ± 0.88	3.39 ± 1.03	0.49
PL (mmol/L)	3.07 ± 0.58	3.00 ± 0.51	0.58
NEFA (mmol/L)	0.52 ± 0.29	0.49 ± 0.22	0.67
HDL-C/LDL-C ratio	0.57 ± 0.23	0.49 ± 0.24	0.12
Non HDL-C (mmol/L)	3.79 ± 1.14	4.01 ± 1.18	0.41
Castelli`s risk index	3.37 ± 0.96	4.10 ± 1.80	0.023
Atherogenic index	-0.21 ± 0.29	-0.13 ± 0.33	0.28
Metabolites			
Betaine (µM)	36.32 ± 11.85	47.47 ± 26.93	0.013
Choline (µM)	11.20 ± 2.23	11.70 ± 2.61	0.009
L-carnitine (µM)	35.04 ± 7.76	39.55 ± 7.92	0.017
GBB (µM)	0.87 ± 0.21	0.96 ± 0.22	0.08
TML (µM)	0.45 ± 0.13	0.57 ± 0.22	0.003
TMAO (µM)	6.16 ± 8.11	7.31 ± 7.62	0.54
Acetyl-C, AC2 (µM)	7.28 ± 3.3	7.22 ± 1.82	0.93
Propionyl-C, AC3 (µM)	0.36 ± 0.11	0.39 ± 0.11	0.26
Valeryl-C, AC5 (µM)	0.20 ± 0.61	0.12 ± 0.04	0.52
Octanoyl-C, AC8 (µM)	0.13 ± 0.06	0.14 ± 0.06	0.38
Lauroyl-C, AC12 (µM)	0.06 ± 0.03	0.06 ± 0.03	0.47
Myristoyl-C, AC14 (µM)	0.03 ± 0.01	0.03 ± 0.01	0.09
Palmitoyl-C, AC16 (µM)	0.10 ± 0.03	0.11 ± 0.03	0.07
AC2 /AC8	59.08 ± 21.42	55.40 ± 17.68	0.44

AC2 /AC12	140.14 ± 50.86	135.73 ± 57.80	0.73
AC2 /AC14	293.55 ± 87.37	265.99 ± 85.49	0.18
AC2 /AC16	71.70 ± 22.15	65.24 ± 14.62	0.17
AC3 /AC16	3.83 ± 1.42	3.60 ± 3.12	0.48

Data represent mean ± SD, standard deviation of observed values. CAL > 5mm, patients with at least 2 sites with CAL > 5mm; Age, age in years at the time of clinical examination; BMI, body mass index; PD, probing depth; CAL, clinical attachment level; BoP, percentage of sites with bleeding on probing; PI, percentage of sites with dental plaque; RA debut, age when diagnosed with RA; DAS28, RA disease activity score; VAS, patient global health assessment score on a visual analogue scale; MHAQ, modified health assessment questionnaire; RF, rheumatoid factor; CRP, C-reactive protein; ESR, erythrocyte sedimentation rate; SFA, saturated FA; MUFA, monounsaturated FA; PUFA, polyunsaturated FA; n-3 PUFA, sum of n-3 PUFA; n-6 PUFA, sum of n-6 PUFA; Omega-3 index, sum of wt% EPA, eicosapentaenoic acid, and wt% DHA, docosahexaenoic acid; AA, arachidonic acid; ALA, alpha-linolenic acid; LA, linoleic acid; AIFA index, anti-inflammatory fatty acid index; DNL index, de novo lipogenesis index; DGLA, dihomo-gamma-linolenic acid; AdA, adrenic acid; DPA, docosapentaenoic acid; TG, triglyceride; TC, total cholesterol; HDL-C, high-density lipoprotein cholesterol; LDL-C, low-density lipoprotein cholesterol; PL, phospholipid; NEFA, non-esterified FA; Castelli's risk index, ratio TC/HDL-C; Atherogenic index, log10(TG/HDL-C); GBB, γ-butyrobetaine; TML, trimethyl lysine; TMAO, trimethylamine N-oxide; Acetyl-C (AC2), Acetyl-Carnitine. Analysed with Student t-test: *P*-level <0.05. † n = 74

Supplementary Table S6. Distribution of demographic, behavioral and clinical continuous variables including lipid-, metabolite- and fatty acid (FA) concentrations in rheumatoid arthritis (RA) patients (*N* = 78) grouped by smoking status.

	Smoking status			*P*-value
Variables	Never (*N* = 29)	Former (*N* = 35)	Current (*N* = 14)	
Age (years)	57 ± 12	58 ± 11	54 ± 12	0.49
BMI (kg/m^2)	24 ± 3	28 ± 5	26 ± 3	0.002
Oral data				
Number of teeth	26 ± 2	23 ± 6	25 ± 5	0.033
PD (mm)	2.6 ± 0.2	2.8 ± 0.4	3.1 ± 0.5	0.002
CAL (mm)	2.8 ± 0.3	3.1 ± 0.8	3.5 ± 1.1	0.011
BoP (%)	25 ± 10	31 ± 15	44 ± 18	<0.001
PI (%)	30 ± 14	33 ± 19	37 ± 14	0.41
RA data				
RA debut (age)	45 ± 17	42 ± 12	41 ± 13	0.62
RA duration (years)	13 ± 11	17 ± 12	13 ± 10	0.34
DAS28 (score)	2.8 ± 0.9	3.1 ± 1.3	3.1 ± 0.9	0.63
VAS (score)	25 ± 21	29 ± 20	31 ± 24	0.53
MHAQ (score)	0.28 ± 0.38	0.41 ± 0.38	0.46 ± 0.34	0.27
Laboratory data				
RF (IU/ml) [†]	123 ± 253	240 ± 514	168 ± 199	0.50
CRP (mg/L)	7 ± 8	11 ± 17	7 ± 11	0.39
ESR (mm/hr)	21 ± 14	20 ± 18	18 ± 13	0.83
FA data				
Total FA (mg)	3.16 ± 0.41	3.16 ± 0.66	2.94 ± 0.43	0.38
SFA (wt%)	37.94 ± 2.27	37.39 ± 1.63	37.15 ± 1.96	0.37
MUFA (wt%)	19.83 ± 1.96	21.00 ± 2.93	21.12 ± 2.71	0.14
PUFA (wt%)	39.46 ± 2.70	38.97 ± 2.25	39.00 ± 2.57	0.70
Unidentified FA (wt%)	0.76 ± 0.62	2.64 ± 0.65	2.74 ± 0.67	0.73
n-3 PUFA (wt%)	9.46 ± 2.21	7.99 ± 1.99	7.75 ± 2.36	0.011
n-6 PUFA (wt%)	30.00 ± 2.35	30.99 ± 2.54	31.24 ± 2.51	0.18
n-3/n-6	0.32 ± 0.08	0.26 ± 0.09	0.25 ± 0.09	0.017
Omega-3 index	7.09 ± 2.13	5.70 ± 1.86	5.56 ± 2.06	0.012
AA/EPA	5.90 ± 4.53	8.50 ± 4.70	10.10 ± 5.77	0.019
EPA/ALA	3.99 ± 2.26	2.69 ± 2.35	2.92 ± 1.87	0.07
AA/LA	0.48 ± 0.10	0.50 ± 0.11	0.53 ± 0.11	0.27
AIFA index	843.05 ± 196.02	722.82 ± 177.67	712.295 ± 199.87	0.024
DNL index	1.28 ± 0.23	1.26 ± 0.19	1.28 ± 0.26	0.86
Elongase C18:0/C16:0	0.51 ± 0.05	0.51 ± 0.06	0.50 ± 0.05	0.92
Desaturase D5n-6	8.31 ± 4.15	7.06 ± 2.33	7.01 ± 1.46	0.22
C14:0 (wt%)	0.93 ± 0.42	0.83 ± 0.32	0.82 ± 0.39	0.46
C15:0 (wt%)	0.28 ± 0.08	0.22 ± 0.10	0.23 ± 0.09	0.06
C16:0 (wt%)	23.87 ± 1.75	23.55 ± 1.35	23.50 ± 1.98	0.67

C16:1 (wt%)	1.51 ± 0.48	1.68 ± 0.59	1.72 ± 0.63	0.40
C16:3n-3 (wt%)	0.37 ± 0.13	0.32 ± 0.11	0.33 ± 0.12	0.24
C17:0 (wt%)	0.25 ± 0.11	0.29 ± 0.13	0.30 ± 0.16	0.43
C18:0 (wt%)	12.03 ± 0.98	11.91 ± 1.06	11.70 ± 0.72	0.59
C18:1 (wt%)	17.37 ± 1.75	18.41 ± 2.56	18.52 ± 2.56	0.14
C18:2n-6, LA (wt%)	18.93 ± 2.19	19.04 ± 2.17	18.70 ± 1.84	0.88
C18:3n-3, ALA (wt%)	0.61 ± 0.23	0.64 ± 0.24	0.55 ± 0.27	0.46
C18:4n-3 (wt%)	0.15 ± 0.08	0.14 ± 0.07	0.10 ± 0.10	0.21
C20:0 (wt%)	0.23 ± 0.07	0.26 ± 0.09	0.23 ± 0.08	0.22
C20:1 (wt%)	0.20 ± 0.08	0.18 ± 0.05	0.17 ± 0.05	0.32
C20:2n-6 (wt%)	0.32 ± 0.13	0.32 ± 0.09	0.36 ± 0.16	0.56
C20:3n-6, DGLA (wt%)	1.20 ± 0.37	1.40 ± 0.28	1.44 ± 0.28	0.018
C20:4n-6, AA (wt%)	8.86 ± 1.25	9.35 ± 1.45	9.82 ± 1.61	0.10
C20:4n-3 (wt%)	0.01 ± 0.02	0.01 ± 0.05	0.03 ± 0.06	0.34
C20:5n-3, EPA (wt%)	2.20 ± 1.12	1.49 ± 1.12	1.39 ± 0.92	0.019
C22:0 (wt%)	0.36 ± 0.07	0.34 ± 0.07	0.39 ± 0.09	0.10
C22:4n-6, AdA (wt%)	0.68 ± 0.21	0.86 ± 0.32	0.87 ± 0.31	0.024
C22:5n-6 (wt%)	0.01 ± 0.05	0.03 ± 0.09	0.06 ± 0.13	0.33
C22:5n-3, DPA (wt%)	1.25 ± 0.19	1.17 ± 0.21	1.19 ± 0.27	0.38
C22:6n-3, DHA (wt%)	4.90 ± 1.13	4.21 ± 0.89	4.17 ± 1.18	0.020
C24:1n-9 (wt%)	0.75 ± 0.18	0.73 ± 0.15	0.71 ± 0.15	0.72
Lipids				
TC (mmol/L)	5.50 ± 1.08	5.46 ± 1.39	5.66 ± 1.13	0.87
TG (mmol/L)	1.02 ± 0.40	1.39 ± 1.08	1.12 ± 0.62	0.18
HDL-C (mmol/L)	1.78 ± 0.46	1.58 ± 0.55	1.48 ± 0.34	0.11
LDL-C (mmol/L)	3.23 ± 0.82	3.20 ± 0.99	3.63 ± 0.99	0.33
PL (mmol/L)	3.04 ± 0.46	3.05 ± 0.67	3.02 ± 0.44	0.99
NEFA (mmol/L)	0.50 ± 0.20	0.50 ± 0.33	0.53 ± 0.21	0.96
HDL-C/LDL-C ratio	0.59 ± 0.21	0.55 ± 0.27	0.44 ± 0.16	0.16
Non HDL-C (mmol/L)	3.72 ± 0.91	3.87 ± 1.34	4.18 ± 1.10	0.47
Castelli`s risk index	3.22 ± 0.77	3.83 ± 1.71	4.00 ± 1.15	0.10
Atherogenic index	-0.26 ± 0.24	-0.12 ± 0.34	-0.16 ± 0.31	0.16
Metabolites				
Betaine (µM)	40.30 ± 14.31	41.86 ± 25.41	36.51 ± 9.55	0.69
Choline (µM)	10.19 ± 2.41	11.21 ± 2.64	10.67 ± 1.95	0.26
L-carnitine (µM)	34.61 ± 8.00	37.85 ± 7.56	37.95 ± 9.09	0.22
GBB (µM)	0.90 ± 0.26	0.95 ± 0.19	0.81 ± 0.14	0.14
TML (µM)	0.49 ± 0.17	0.51 ± 0.20	0.46 ± 0.15	0.62
TMAO (µM)	8.90 ± 11.69	5.96 ± 4.35	3.30 ± 1.42	0.08
Acetyl-C, AC2 (µM)	7.31 ± 3.37	7.42 ± 2.62	6.74 ± 2.06	0.75
Propionyl-C, AC3 (µM)	0.34 ± 0.09	0.40 ± 0.11	0.36 ± 0.13	0.06
Valeryl-C, AC5 (µM)	0.11 ± 0.04	0.12 ± 0.06	0.41 ± 1.15	0.12
Octanoyl-C, AC8 (µM)	0.13 ± 0.06	0.14 ± 0.06	0.13 ± 0.04	0.61
Lauroyl-C, AC12 (µM)	0.06 ± 0.03	0.06 ± 0.03	0.05 ± 0.02	0.29
Myristoyl-C, AC14 (µM)	0.03 ± 0.01	0.03 ± 0.01	0.02 ± 0.01	0.39
Palmitoyl-C, AC16 (µM)	0.10 ± 0.02	0.11 ± 0.04	0.10 ± 0.02	0.45

AC2 /AC8	61.09 ± 22.95	55.27 ± 16.76	57.08 ± 22.05	0.52
AC2 /AC12	140.86 ± 56.75	131.35 ± 42.83	151.79 ± 68.13	0.46
AC2 /AC14	280.09 ± 86.51	281.74 ± 84.27	295.82 ± 100.56	0.85
AC2 /AC16	71.84 ± 25.32	68.51 ± 15.31	66.49 ± 18.25	0.68
AC3 /AC16	3.48 ± 1.20	4.02 ± 1.52	3.60 ± 1.14	0.26

Data represent mean ± SD, standard deviation of observed values. Age, age in years at the time of clinical examination; BMI, body mass index; PD, probing depth; CAL, clinical attachment level; BoP, bleeding on probing; PI, plaque index; RA debut, age when diagnosed with RA; DAS28, RA disease activity score; VAS, patient global health assessment score on a visual analogue scale; MHAQ, modified health assessment questionnaire; RF, rheumatoid factor; CRP, C-reactive protein; ESR, erythrocyte sedimentation rate; SFA, saturated FA; MUFA, monounsaturated FA; PUFA, polyunsaturated FA; n-3 PUFA, sum of PUFA n-3; n-6 PUFA, sum of PUFA n-6; Omega-3 index, sum of wt% EPA, eicosapentaenoic acid, and wt% DHA, docosahexaenoic acid; AA, arachidonic acid; ALA, alpha-linolenic acid; LA, linoleic acid; AIFA index, anti-inflammatory fatty acid index; DNL index, de novo lipogenesis index; DGLA, dihomo-gamma-linolenic acid; AdA, adrenic acid; DPA, docosapentaenoic acid; TC, total cholesterol; TG, triglyceride; HDL-C, high-density lipoprotein cholesterol; LDL-C, low-density lipoprotein cholesterol; PL, phospholipid; NEFA, non-esterified FA; Castelli`s risk index, ratio TC/HDL-C; Atherogenic index, log10(TG/HDL-C); GBB, γ-butyrobetaine; TML, trimethyl lysine; TMAO, trimethylamine N-oxide; Acetyl-C, Acetyl-Carnitine. Analysed with one-way ANOVA: *P*-level <0.05. † n = 74

Supplementary Table S7. Distribution of demographic, behavioral and clinical continuous variables including lipid-, metabolite- and fatty acid (FA) concentrations in rheumatoid arthritis (RA) patients (*N* = 78) grouped by trimethylamine N-oxide (TMAO) percentiles.

Variables	TMAO percentiles<br>1st percentile (*N* = 39)	 2nd percentile (*N* = 39)	*P*-value
Age (years)	55 ± 12	59 ± 11	0.07
BMI (kg/m^2)	26 ± 5	26 ± 4	0.66
Oral data			
Number of teeth	25 ± 4	24 ± 6	0.24
PD (mm)	2.7 ± 0.3	2.8 ± 0.5	0.06
CAL (mm)	2.9 ± 0.5	3.2 ± 1.0	0.11
BoP (%)	30 ± 14	33 ± 18	0.39
PI (%)	30 ± 11	36 ± 20	0.14
RA data			
RA debut (age)	42 ± 13	45 ± 15	0.35
RA duration (years)	14 ± 12	15 ± 11	0.71
DAS28 (score)	2.9 ± 0.9	3.1 ± 1.3	0.39
VAS (score)	27 ± 19	29 ± 22	0.64
MHAQ (score)	0.38 ± 0.39	0.36 ± 0.37	0.80
Laboratory data			
RF (IU/ml) †	249 ± 500	121 ± 218	0.15
CRP (mg/L)	6 ± 8	11 ± 17	0.07
ESR (mm/hr)	17 ± 14	23 ± 17	0.07
FA data			
Total FA (mg)	3.17 ± 0.62	3.07 ± 0.45	0.41
SFA (wt%)	37.66 ± 1.70	37.45 ± 2.18	0.65
MUFA (wt%)	21.15 ± 2.75	20.02 ± 2.35	0.05
PUFA (wt%)	38.44 ± 2.62	39.88 ± 2.08	0.009
Unidentified FA (wt%)	2.76 ± 0.64	2.65 ± 0.64	0.45
n-3 PUFA (wt%)	8.06 ± 2.29	8.92 ± 2.15	0.09
n-6 PUFA (wt%)	30.38 ± 2.87	30.96 ± 2.04	0.31
n-3/n-6	0.27 ± 0.10	0.29 ± 0.08	0.33
Omega-3 index	5.79 ± 2.14	6.60 ± 5.96	0.09
AA/EPA	8.98 ± 5.61	6.66 ± 4.14	0.041
EPA/ALA	3.01 ± 2.53	3.43 ± 2.04	0.42
AA/LA	0.52 ± 0.11	0.48 ± 0.09	0.10
AIFA index	728.82 ± 201.49	802.44 ± 184.81	0.10
DNL index	1.30 ± 0.23	1.24 ± 0.19	0.16
Elongase C18:0/C16:0	0.51 ± 0.05	0.50 ± 0.05	0.34
Desaturase D5n-6	7.25 ± 2.32	7.78 ± 3.67	0.45
C14:0 (wt%)	0.89 ± 0.38	0.84 ± 0.36	0.60
C15:0 (wt%)	0.24 ± 0.10	0.25 ± 0.10	0.43
C16:0 (wt%)	23.62 ± 1.58	23.70 ± 1.67	0.84
C16:1 (wt%)	1.69 ± 0.61	1.57 ± 0.50	0.33

C16:3n-3 (wt%)	0.34 ± 0.12	0.34 ± 0.12	0.89
C17:0 (wt%)	0.29 ± 0.14	0.27 ± 0.11	0.59
C18:0 (wt%)	12.02 ± 0.89	11.81 ± 1.04	0.34
C18:1 (wt%)	18.57 ± 2.45	17.52 ± 2.08	0.046
C18:2n-6, LA (wt%)	18.46 ± 2.24	19.41 ± 1.86	0.046
C18:3n-3, ALA (wt%)	0.60 ± 0.22	0.63 ± 0.26	0.53
C18:4n-3 (wt%)	0.13 ± 0.08	0.13 ± 0.09	0.93
C20:0 (wt%)	0.24 ± 0.08	0.25 ± 0.09	0.68
C20:1 (wt%)	0.18 ± 0.06	0.19 ± 0.06	0.28
C20:2n-6 (wt%)	0.32 ± 0.14	0.33 ± 0.10	0.82
C20:3n-6, DGLA (wt%)	1.37 ± 0.33	1.29 ± 0.33	0.28
C20:4n-6, AA (wt%)	9.37 ± 1.62	9.14 ± 1.23	0.48
C20:4n-3 (wt%)	0.01 ± 0.04	0.01 ± 0.05	0.77
C20:5n-3, EPA (wt%)	1.56 ± 1.15	1.91 ± 1.10	0.17
C22:0 (wt%)	0.37 ± 0.07	0.34 ± 0.07	0.07
C22:4n-6, AdA (wt%)	0.82 ± 0.34	0.76 ± 0.24	0.37
C22:5n-6 (wt%)	0.03 ± 0.08	0.03 ± 0.09	0.84
C22:5n-3, DPA (wt%)	1.20 ± 0.24	1.21 ± 0.19	0.93
C22:6n-3, DHA (wt%)	4.23 ± 1.14	4.69 ± 0.98	0.06
C24:1n-9 (wt%)	0.72 ± 0.18	0.74 ± 0.15	0.57
Lipids			
TG (mmol/L)	1.30 ± 1.00	1.10 ± 0.58	0.28
TC (mmol/L)	5.56 ± 1.37	5.46 ± 1.06	0.72
HDL-C (mmol/L)	1.61 ± 0.48	1.67 ± 0.51	0.57
LDL-C (mmol/L)	3.33 ± 0.97	3.25 ± 0.90	0.73
PL (mmol/L)	3.09 ± 0.63	2.99 ± 0.47	0.40
NEFA (mmol/L)	0.55 ± 0.33	0.46 ± 0.18	0.15
HDL-C/LDL-C ratio	0.52 ± 0.22	0.56 ± 0.25	0.46
Non HDL-C (mmol/L)	3.95 ± 1.25	3.79 ± 1.06	0.53
Castelli`s risk index	3.76 ± 1.56	3.51 ± 1.12	0.43
Atherogenic index	-0.14 ± 0.32	-0.21 ± 0.29	0.31
Metabolites			
Betaine (µM)	37.51 ± 11.89	43.13 ± 24.46	0.20
Choline (µM)	10.38 ± 2.24	11.09 ± 2.65	0.20
L-carnitine (µM)	36.19 ± 6.95	37.13 ± 9.11	0.61
GBB (µM)	0.84 ± 0.19	0.97 ± 0.23	0.011
TML (µM)	0.43 ± 0.13	0.56 ± 0.20	<0.001
TMAO (µM)	2.61 ± 0.89	10.53 ± 9.68	<0.001
Acetyl-C, AC2 (µM)	7.30 ± 3.28	7.21 ± 2.31	0.89
Propionyl-C, AC3 (µM)	0.35 ± 0.09	0.39 ± 0.12	0.15
Valeryl-C, AC5 (µM)	0.23 ± 0.69	0.12 ± 0.04	0.33
Octanoyl-C, AC8 (µM)	0.14 ± 0.06	0.13 ± 0.05	0.41
Lauroyl-C, AC12 (µM)	0.06 ± 0.03	0.06 ± 0.03	0.41
Myristoyl-C, AC14 (µM)	0.03 ± 0.01	0.03 ± 0.01	0.98
Palmitoyl-C, AC16 (µM)	0.10 ± 0.03	0.11 ± 0.03	0.68
AC2 /AC8	56.73 ± 23.21	58.79 ± 16.72	0.66
AC2 /AC12	130.20 ± 48.34	146.92 ± 56.91	0.17
AC2 /AC14	278.47 ± 82.14	288.84 ± 92.70	0.60

AC2 /AC16	69.57 ± 20.29	69.19 ± 19.81	0.93
AC3 /AC16	3.62 ± 1.24	3.87 ± 1.46	0.42

Data represent mean ± SD, standard deviation of observed values. TMAO percentiles, TMAO values categorized into lowest (median 2.66, range 0.67 – 4.21) and highest percentile (median 6.84, range 4.29 – 44.28); Age, age in years at the time of clinical examination; BMI, body mass index; PD, probing depth; CAL, clinical attachment level; BoP, bleeding on probing; PI, plaque index; RA debut, age when diagnosed with RA; DAS28, RA disease activity score; VAS, patient global health assessment score on a visual analogue scale; MHAQ, modified health assessment questionnaire; RF, rheumatoid factor; CRP, C-reactive protein; ESR, erythrocyte sedimentation rate; SFA, saturated FA; MUFA, monounsaturated FA; PUFA, polyunsaturated FA; n-3 PUFA, sum of n-3 PUFA; n-6 PUFA, sum of n-6 PUFA; Omega-3 index, sum of wt% EPA, eicosapentaenoic acid, and wt% DHA, docosahexaenoic acid; AA, arachidonic acid; ALA, alpha-linolenic acid; LA, linoleic acid; AIFA index, anti-inflammatory fatty acid index; DNL index, de novo lipogenesis index; DGLA, dihomo-gamma-linolenic acid; AdA, adrenic acid, DPA, docosapentaenoic acid; TG, triglyceride; TC, total cholesterol; HDL-C, high-density lipoprotein cholesterol; LDL-C, low-density lipoprotein cholesterol; PL, phospholipid; NEFA, non-esterified FA; Castelli`s risk index, TC/HDL-C; Atherogenic index, log10(TG/HDL-C); GBB, γ-butyrobetaine; TML, trimethyl lysine; TMAO, trimethylamine N-oxide; Acetyl-C (AC2), Acetyl-Carnitine. Analysed with Student t-test: *P*-level <0.05. † n = 74

Supplementary Table S8. Distribution of demographic, behavioral and clinical continuous variables including lipid-, metabolite- and fatty acid (FA) concentrations in rheumatoid arthritis (RA) patients (*N* = 78) grouped by Omega-3 index tertiles.

Variables	Omega-3 tertiles			*P*-value
	1st Tertile (*N* = 26)	2nd Tertile (*N* = 26)	3rd Tertile (*N* = 26)	
Age (years)	51 ± 12	60 ± 10	61 ± 10	0.001
BMI (kg/m^2)	27 ± 4	27 ± 5	24 ± 3	0.020
Oral data				
Number of teeth	26 ± 3	23 ± 6	25 ± 5	0.08
PD (mm)	2.8 ± 0.3	2.9 ± 0.5	2.7 ± 0.3	0.26
CAL (mm)	2.9 ± 0.5	3.3 ± 1.1	2.9 ± 0.4	0.13
BoP (%)	35 ± 16	32 ± 14	27 ± 16	0.20
PI (%)	32 ± 11	36 ± 23	30 ± 13	0.33
RA data				
RA debut (age)	38 ± 12	49 ± 12	43 ± 17	0.019
RA duration (years)	14 ± 9	12 ± 10	19 ± 13	0.07
DAS28 (score)	3.0 ± 0.8	2.9 ± 1.3	3.1 ± 1.2	0.92
VAS (score)	35 ± 18	22 ± 22	26 ± 21	0.06
MHAQ (score)	0.44 ± 0.39	0.32 ± 0.39	0.35 ± 0.36	0.54
Laboratory data				
RF (IU/ml) [†]	206 ± 411	172 ± 408	170 ± 342	0.93
CRP (mg/L)	7 ± 10	9 ± 13	10 ± 17	0.58
ESR (mm/hr)	15 ± 12	21 ± 17	23 ± 17	0.09
FA data				
Total FA (mg)	3.19 ± 0.63	3.18 ± 0.51	2.99 ± 0.47	0.32
SFA (wt%)	37.51 ± 1.79	38.11 ± 2.02	37.40 ± 1.96	0.18
MUFA (wt%)	22.16 ± 2.64	20.35 ± 2.36	19.25 ± 1.96	<0.001
PUFA (wt%)	38.10 ± 2.17	38.79 ± 2.09	40.58 ± 2.49	<0.001
Unidentified FA (wt%)	2.59 ± 0.71	2.75 ± 0.57	2.77 ± 0.64	0.55
n-3 PUFA (wt%)	6.31 ± 0.83	8.15 ± 0.61	11.01 ± 1.67	<0.001
n-6 PUFA (wt%)	31.79 ± 2.05	30.64 ± 2.48	29.56 ± 2.48	0.004
n-3/n-6	0.20 ± 0.03	0.27 ± 0.04	0.38 ± 0.08	<0.001
Omega-3 index	4.13 ± 0.55	5.89 ± 0.57	8.56 ± 1.58	<0.001
AA/EPA	12.94 ± 4.50	7.16 ± 2.68	3.36 ± 1.48	<0.001
EPA/ALA	1.60 ± 0.95	2.94 ± 1.51	5.12 ± 2.53	<0.001
AA/LA	0.51 ± 0.10	0.52 ± 0.11	0.46 ± 0.10	0.14
AIFA index	571.25 ± 57.06	740.64 ± 63.20	985.01 ± 143.54	<0.001
DNL index	1.23 ± 0.18	1.32 ± 0.24	1.27 ± 0.21	0.31
Elongase C18:0/C16:0	0.51 ± 0.05	0.51 ± 0.06	0.51 ± 0.05	0.88
Desaturase D5n-6	6.79 ± 1.60	7.13 ± 1.97	8.63 ± 4.52	0.07
C14:0 (wt%)	0.94 ± 0.44	0.87 ± 0.37	0.78 ± 0.27	0.26

C15:0 (wt%)	0.23 ± 0.10	0.22 ± 0.08	0.28 ± 0.10	0.08
C16:0 (wt%)	23.41 ± 1.52	24.03 ± 1.83	23.54 ± 1.46	0.35
C16:1 (wt%)	1.81 ± 0.61	1.60 ± 0.60	1.46 ± 0.40	0.07
C16:3n-3 (wt%)	0.34 ± 0.11	0.32 ± 0.10	0.35 ± 0.13	0.69
C17:0 (wt%)	0.28 ± 0.13	0.28 ± 0.13	0.27 ± 0.13	0.95
C18:0 (wt%)	11.69 ± 0.92	12.13 ± 1.04	11.93 ± 0.94	0.26
C18:1 (wt%)	19.51 ± 2.28	17.88 ± 2.07	16.75 ± 1.76	<0.001
C18:2n-6, LA (wt%)	19.31 ± 1.89	18.60 ± 2.18	18.91 ± 2.22	0.48
C18:3n-3, ALA (wt%)	0.61 ± 0.28	0.59 ± 0.24	0.64 ± 0.21	0.76
C18:4n-3 (wt%)	0.15 ± 0.08	0.13 ± 0.08	0.21 ± 0.09	0.49
C20:0 (wt%)	0.24 ± 0.08	0.24 ± 0.08	0.25 ± 0.08	0.82
C20:1 (wt%)	0.17 ± 0.04	0.16 ± 0.05	0.23 ± 0.06	<0.001
C20:2n-6 (wt%)	0.34 ± 0.14	0.31 ± 0.12	0.32 ± 0.11	0.73
C20:3n-6, DGLA (wt%)	1.47 ± 0.29	1.39 ± 0.28	1.13 ± 0.33	<0.001
C20:4n-6, AA (wt%)	9.63 ± 1.38	9.51 ± 1.45	8.63 ± 1.30	0.021
C20:4n-3 (wt%)	0	0.02 ± 0.05	0.02 ± 0.06	0.30
C20:5n-3, EPA (wt%)	0.82 ± 0.25	1.44 ± 0.39	2.94 ± 1.12	<0.001
C22:0 (wt%)	0.35 ± 0.08	0.35 ± 0.07	0.36 ± 0.08	0.82
C22:4n-6, AdA (wt%)	1.00 ± 0.24	0.80 ± 0.27	0.57 ± 0.18	<0.001
C22:5n-6 (wt%)	0.05 ± 0.12	0.04 ± 0.09	0	0.14
C22:5n-3, DPA (wt%)	1.08 ± 0.19	1.21 ± 0.16	1.33 ± 0.22	<0.001
C22:6n-3, DHA (wt%)	3.31 ± 0.46	4.45 ± 0.37	5.62 ± 0.69	<0.001
C24:1n-9 (wt%)	0.67 ± 0.14	0.71 ± 0.16	0.82 ± 0.16	0.003
Lipids				
TC (mmol/L)	5.34 ± 1.56	5.59 ± 0.93	5.60 ± 1.12	0.67
TG (mmol/L)	1.47 ± 1.14	1.24 ± 0.57	0.90 ± 0.52	0.037
HDL-C (mmol/L)	1.44 ± 0.39	1.57 ± 0.54	1.92 ± 0.43	<0.001
LDL-C (mmol/L)	3.24 ± 1.16	3.40 ± 0.71	3.23 ± 0.90	0.77
PL (mmol/L)	3.01 ± 0.72	3.07 ± 0.43	3.05 ± 0.49	0.92
NEFA (mmol/L)	0.52 ± 0.36	0.48 ± 0.20	0.52 ± 0.21	0.81
HDL-C/LDL-C ratio	0.51 ± 0.24	0.48 ± 0.20	0.64 ± 0.25	0.025
Non HDL-C (mmol/L)	3.91 ± 1.51	4.03 ± 0.81	3.68 ± 1.05	0.55
Castelli`s risk index	3.86 ± 1.16	4.02 ± 1.78	3.02 ± 0.74	0.015
Atherogenic index	-0.05 ± 0.26	-0.11 ± 0.29	-0.37 ± 0.27	<0.001
Metabolites				
Betaine (µM)	41.39 ± 14.01	40.80 ± 27.69	38.77 ± 13.44	0.88
Choline (µM)	10.81 ± 2.79	11.57 ± 2.12	9.83 ± 2.20	0.037
L-carnitine (µM)	38.13 ± 7.52	36.87 ± 8.38	34.98 ± 8.27	0.37
GBB (µM)	0.91 ± 0.21	0.92 ± 0.22	0.90 ± 0.23	0.95
TML (µM)	0.47 ± 0.14	0.48 ± 0.21	0.52 ± 0.18	0.63
TMAO (µM)	4.57 ± 4.06	4.97 ± 2.79	10.18 ± 12.16	0.015
Acetyl-C, AC2 (µM)	7.12 ± 2.68	6.91 ± 2.44	7.74 ± 3.31	0.55
Propionyl-C, AC3 (µM)	0.37 ± 0.08	0.38 ± 0.11	0.37 ± 0.13	0.91
Valeryl-C, AC5 (µM)	0.12 ± 0.06	0.11 ± 0.04	0.28 ± 0.84	0.41
Octanoyl-C, AC8 (µM)	0.14 ± 0.07	0.13 ± 0.05	0.14 ± 0.05	0.61
Lauroyl-C, AC12 (µM)	0.06 ± 0.03	0.06 ± 0.03	0.06 ± 0.02	0.85

Myristoyl-C, AC14 (µM)	0.03 ± 0.01	0.03 ± 0.01	0.03 ± 0.01	0.89
Palmitoyl-C, AC16 (µM)	0.11 ± 0.03	0.11 ± 0.03	0.10 ± 0.02	0.48
AC2 /AC8	55.41 ± 22.06	57.31 ± 17.45	60.57 ± 21.00	0.65
AC2 /AC12	142.01 ± 66.52	134.22 ± 37.38	139.45 ± 53.27	0.87
AC2 /AC14	286.60 ± 94.42	279.15 ± 86.34	285.21 ± 83.66	0.95
AC2 /AC16	67.36 ± 18.01	63.86 ± 16.48	76.94 ± 23.06	0.048
AC3 /AC16	3.73 ± 1.13	3.66 ± 1.24	3.84 ± 1.67	0.90

Data represent mean ± SD, standard deviation of observed values. Omega-3 tertiles, 1st Tertile (median 4.04, range 3.03 – 4.95), 2nd Tertile (median 5.81, range 5.08 – 6.75), 3rd Tertile (median 7.66, range 6.86 – 12.62); Age, age in years at the time of clinical examination; BMI, body mass index; PD, probing depth; CAL, clinical attachment level; BoP, percentage of sites with bleeding on probing; PI, percentage of sites with dental plaque; RA debut, age when diagnosed with RA; DAS28, RA disease activity score; VAS, patient global health assessment score on a visual analogue scale; MHAQ, modified health assessment questionnaire; RF, rheumatoid factor; CRP, C-reactive protein; ESR, erythrocyte sedimentation rate; SFA, saturated FA; MUFA, monounsaturated FA; PUFA, polyunsaturated FA; n-3 PUFA, sum of n-3 PUFA; n-6 PUFA, sum of n-6 PUFA; Omega-3 index, sum of wt% EPA, eicosapentaenoic acid, and wt% DHA, docosahexaenoic acid; AA, arachidonic acid; ALA, alpha-linolenic acid; LA, linoleic acid; AIFA index, anti-inflammatory fatty acid index; DNL index, de novo lipogenesis index; DGLA, dihomo-gamma-linolenic acid; AdA, adrenic acid; DPA, docosapentaenoic acid; TC, total cholesterol; TG, triglyceride; HDL-C, high-density lipoprotein cholesterol; LDL-C, low-density lipoprotein cholesterol; PL, phospholipid; NEFA, non-esterified FA; Castelli`s risk index, ratio TC/HDL-C; Atherogenic index, log10(TG/HDL-C); GBB, γ-butyrobetaine; TML, trimethyl lysine; TMAO, trimethylamine N-oxide; Acetyl-C (AC2), Acetyl-Carnitine. Analysed with one-way ANOVA: *P*-level <0.05. † n = 74
